# Dynamics of Endoplasmic Reticulum Stress Responses in Cells with Different Endoplasmic Reticulum Volumes and Senescence States

**DOI:** 10.3390/cimb48101027

**Published:** 2026-10-04

**Authors:** Aleksandra V. Mishina, Alexey. Yu. Pas’ko, Dmitry V. Kolesov, Lyudmila V. Kovalenko, Danila M. Zaychenko, Sergey G. Morozov, Alexey A. Moskovtsev

**Affiliations:** 1 Cellular Stress Laboratory, FSBI Institute of General Pathology and Pathophysiology, 125315 Moscow, Russia; aleksandramishin@gmail.com (A.V.M.);; 2 Medical Institute, Surgut State University, Yugra, 628408 Surgut, Russia; 3 Russian Medical Academy of Postdegree Education, 125993 Moscow, Russia

**Keywords:** unfolded protein response (UPR), endoplasmic reticulum (ER) stress, cellular senescence, cell geometry and volume, temporal dynamics, gene response time, mathematical modeling, scanning ion-conductance microscopy (SICM)

## Abstract

The Unfolded Protein Response (UPR) determines cell fate under endoplasmic reticulum (ER) stress, yet its temporal dynamics across different cell types and senescence states re-main poorly understood. In this study, we investigated UPR kinetics in endothelial and T-lymphoblastoid cell lines with distinct ER volumes, as well as in senescent FRSN cells undergoing ER expansion. Variations in ER volume were confirmed via confocal microscopy and flow cytometry. Using the ‘gene response time’ metric, we demonstrated experimentally and through mathematical modeling that the BiP response kinetics closely correlate with variations in ER volume. Applying this metric to presenescent FRSN cells with a significantly expanded ER, we found that their ER stress response machinery maintained a response time similar to that of exponentially growing FRSN cells, but exhibited a lower amplitude and a prolonged lag phase, leading to chronic ER stress signaling such as a protracted XBP1(S) expression profile. Scanning ion-conductance microscopy (SICM) revealed a significant decrease in the stiffness of the near-surface layer of presenescent cells, reflecting a rarefaction of the actin cortex and possibly cytoplasmic dilution. We propose that this structural remodeling could be associated with active intracellular transport impairment that weakens the temporal ‘tethering’ of the proteome to the genome upon cellular senescence. Consequently, the stress response machinery fails to scale proportionally with cell enlargement during senescence, creating a state of ‘functional dilution’. This prevents enlarged senescent cells from mounting a scaled response, resulting in prolonged stress signaling and increased vulnerability to proteotoxic stress.

## 1. Introduction

The endoplasmic reticulum (ER) stress response is a compartmentalized form of the cellular stress response that usually involves the sensing of unfolded/misfolded proteins in the ER lumen, followed by the processing and transmission of this information to the nucleus and other cellular compartments [1,2]. These functions are realized by a system of signaling pathways known as the Unfolded Protein Response (UPR) [3,4]. UPR activation results in adaptive changes in the ER and in ER-associated structures [5] which aim to reduce the load of unfolded proteins through a number of processes, including translational repression [6], selective mRNA degradation by regulated IRE1α-dependent decay (RIDD) [7], transcriptionally mediated increases in folding capacity, and enhanced ER-associated protein degradation.

The key sensors of the UPR that maintain homeostatic control within the ER are the transmembrane proteins IRE1 (encoded by *ERN1*), PERK (encoded by *EIF2AK3*), and ATF6. These sensors share a common structural organization consisting of three functional domains: a luminal domain that detects protein folding overload/misfolding by sensing unfolded proteins; a transmembrane domain that anchors the protein in the ER membrane; and a cytoplasmic effector domain that modulates downstream cellular signaling, transcription, and translation [4,8]. The accumulation of proteins with non-native conformations within the ER lumen triggers the dissociation of the chaperone BiP from the luminal domain of IRE1, prompting the oligomerization of the IRE1 kinase and the subsequent trans-autophosphorylation of its cytoplasmic domains. This autophosphorylation event activates the endoribonuclease (RNase) domain of IRE1, ultimately leading to the unconventional splicing of XBP1 mRNA [9]. Following translation, spliced XBP1 upregulates the transcription of ER chaperones, folding accessory proteins, components of the ER-associated degradation (ERAD) system, and target genes involved in UPR feedback loops [8].

Due to the presence of intra- and intermolecular disulfide bonds formed between two conserved cysteine residues within its luminal domain, ATF6 resides in the ER membrane under basal conditions as oxidized monomers, dimers, or oligomers [10]. Upon ER stress induction, ATF6 undergoes reduction and is transported as reduced monomers [10] to the Golgi apparatus via COPII-coated vesicles. In the Golgi, it is sequentially cleaved by site-1 protease (S1P) and site-2 protease (S2P), releasing a 50 kDa cytosolic fragment that functions as an active bZIP domain-containing transcription factor [11]. This fragment translocates to the nucleus, where it binds to the endoplasmic reticulum stress response element (ERSE) motif and interacts with the general transcription factor NF-Y to activate the transcription of ER chaperones, such as BiP, folding accessory proteins, and UPR target genes, including XBP1 and CHOP [11,12]. Notably, reduction alone is insufficient for ATF6 activation; these disulfide bonds are reduced not only by reducing agents like dithiothreitol but also under the influence of tunicamycin, another canonical ER stress inducer that inhibits glycosylation [10]. The activation of ATF6 can be viewed as an indirect process governed by BiP availability, where the loss of BiP binding serves as the primary switch for downstream proteolytic processing. ATF6 may function as an ER redox state sensor. Given its transport to the Golgi via COPII vesicles [13], ATF6 could also potentially serve as a biological quality control metric for the functionality of the ER-to-Golgi secretory pathway, particularly because disruptions in ER–Golgi trafficking can inherently trigger ER overload and stress.

Under ER stress, following the dissociation of the chaperone BiP from its luminal domain, the PERK kinase oligomerizes, autophosphorylates, and subsequently phosphorylates the translation initiation factor eIF2α. This phosphorylation event results in the global attenuation of cap-dependent protein synthesis [3,14]. However, phosphorylated eIF2α selectively activates the translation of a subset of mRNAs containing multiple upstream open reading frames (uORFs) located before the main coding sequence. This mechanism drives the translation of the transcription factor ATF4 mRNA, whose protein product upregulates the expression of genes involved in amino acid metabolism, redox homeostasis, apoptosis, and the ER-associated degradation (ERAD) system [14,15,16].

The stress responses of different cell types in metazoans are generally non-specific and conserved. However, distinct morphological and functional variations across cell types can dictate specific quantitative parameters, leading to cell type-specific kinetic profiles. Because cellular stress is a critical driver of numerous pathological processes, its temporal dynamics—as well as the synchrony or asynchrony of these responses within tissues and the whole organism—are pivotal in shaping disease pathogenesis. While significant focus has been placed on the mathematical modeling and experimental dynamics of the heat shock response [12], our understanding of endoplasmic reticulum (ER) stress dynamics remains predominantly qualitative. Consequently, extrapolating kinetic outcomes to diverse cellular contexts remains challenging, despite an expanding knowledge of mammalian ER stress response machinery and the availability of several mathematical models [17,18,19,20].

To evaluate cell type-specific parameters in the ER stress response, we compared signaling dynamics across human cell lines with distinct basal ER protein-folding capacities. The most prominent morphological manifestation of this difference is reflected in their respective ER volumes. We propose that the overall dimensions of both the cells and their organelle compartments, in conjunction with their basal folding capacity, shape the temporal dynamics of cellular stress responses.

Basal ER protein folding capacity depends on the scale of the cellular ER protein-folding system, which consists of chaperones, folding enzymes, and protein quality control and degradation machinery [21]. The ER has two major chaperone systems: lectin-like chaperones calnexin/calreticulin and chaperones that interact with proteins containing hydrophobic residues [22]. The most abundant member of the latter group is the BiP protein (GRP78, HSPA5), which is an ER-resident Hsp70 family member. BiP is the master regulator of protein folding and oligomerization in the ER [23]. It appears to be the key chaperone that is monitored by the cell to sense ER stress [22]. Under conditions in which N-linked glycosylation is blocked, BiP serves as a backup for calnexin and calreticulin in retaining unfolded proteins in the ER [24]. Therefore, as a collector of unfolded proteins, the BiP protein is an integral indicator of the recovery of ER folding capacity during ER stress. BiP also performs many other functions including maintaining the permeability barrier of the ER by sealing the luminal side of inactive translocons, facilitating the translocation of growing nascent chains into the ER lumen, regulating the aggregation of non-native polypeptides, contributing to ER calcium homeostasis, and participating in the transfer of terminally misfolded ER proteins into the cytosol for subsequent degradation [23].

The IRE1α-XBP1 pathway is a core and evolutionarily ancient component of the UPR. The RNase activity of IRE1α is transient during ER stress, and the IRE1α signaling pathway is attenuated at a particular time point [25,26]. The time period during which IRE1α is active was previously shown to vary between different cell types [27], but the nature of these differences remains unclear. BiP represses IRE1α signaling through its association with the IRE1α luminal domain [28], being a luminal attenuator of IRE1α activity along with the recently reported protein disulfide isomerase A6 [29]. Consequently, BiP decreases IRE1α endoribonuclease activity, promoting the dissociation of its clusters [26]. The temporal control of IRE1α-XBP1 signaling exerts a great influence on the balance between pro- and anti-apoptotic signals in ER-stressed cells [30,31].

To investigate the cell type-specific dynamic features of ER stress responses, we examined IRE1α-XBP1 signaling and the temporal expression patterns of UPR genes. We utilized cell lines with distinct basal ER folding capacities, which are morphologically manifested as variations in ER volume (large in endothelial EA.hy926 cells and small in Jurkat T-cells). We demonstrated consistency between the experimental data and our mathematical model of the UPR. Furthermore, given that variations in ER volume directly shape the specific dynamics of ER stress responses, we investigated changes in the temporal expression profiles of UPR genes during replicative senescence in fibroblasts—a process inherently characterized by a marked increase in cell size accompanied by ER volume expansion.

## 2. Materials and Methods

### 2.1. Reagents

A pharmacological inhibitor of IRE1α (IRE1α inhibitor I, STF-083010) was obtained from Calbiochem-EMD Chemicals Inc. (Darmstadt, Germany) and dissolved in dimethylsulfoxide (Sigma-Aldrich, St. Louis, MO, USA). DL-dithiothreitol was obtained from Sigma-Aldrich (St. Louis, MO, USA). A rabbit polyclonal antibody against BiP(ab21685) was purchased from Abcam (Cambridge, UK). A fluorescein-labeled goat anti-rabbit secondary antibody (IgG) (02-15-06) was procured from KPL (Gaithersburg, MD, USA). PCR primers were synthesized by DNA-Synthesis (Moscow, Russia).

### 2.2. Cell Cultures and Senescence Induction

The Jurkat cell line was obtained from the Russian Cell Culture Collection (Institute of Cytology, Russian Academy of Sciences, Saint Petersburg, Russia) and maintained in RPMI-1640 medium (Life Technologies, Grand Island, NY, USA) supplemented with 10% (*v*/*v*) fetal bovine serum (FBS; Life Technologies, Grand Island, NY, USA) and 50 µg/mL gentamicin (Life Technologies). Human EA.hy926 endothelial cells were kindly provided by Dr. C.J. Edgell (University of North Carolina, Chapel Hill, NC, USA) and cultured in Dulbecco’s Modified Eagle’s Medium (DMEM; Life Technologies) supplemented with 10% (*v*/*v*) FBS, 1% non-essential amino acids (Paneko, Moscow, Russia), 2 mM glutamine (Paneko), HAT supplement (Life Technologies), and 50 µg/mL gentamicin.

The FRSN fibroblast-like cell line was obtained from the Russian Collection of Cell Cultures of Vertebrates (Institute of Cytology RAS, Saint Petersburg, Russia) [32] and cultured in DMEM supplemented with 10% (*v*/*v*) FBS. Analysis was performed using fibroblasts in the exponential growth phase (12th population doubling) and presenescent fibroblasts at late passages (50th population doubling). The latter were close to the Hayflick limit and exhibited a significantly reduced doubling rate. All cells were maintained at 37 °C in a humidified atmosphere containing 5% CO_2_.

### 2.3. Senescence-Associated β-Galactosidase Assay

To confirm the senescent phenotype, senescence-associated beta-galactosidase (SA-β-gal) activity was evaluated using bright-field microscopy. Cells were incubated with X-gal substrate in a staining buffer containing magnesium and calcium chlorides, potassium hexacyanoferrates (II and III), citric acid, and sodium phosphate dibasic at pH 6.0.

### 2.4. Experimental Treatments

To induce ER stress, cells were treated with various concentrations of dithiothreitol (DTT; Sigma-Aldrich, St. Louis, MO, USA) for specified time intervals, as reported previously [30,33].

To investigate the role of the IRE1α pathway, the specific small-molecule inhibitor STF 083010 was used to selectively block the endoribonuclease activity of IRE1α without affecting its kinase domain. Cells were pre-incubated with the inhibitor STF 083010 at a final concentration of 60 µM before ER stress induction. Control groups received an equivalent volume of vehicle (DMSO).

### 2.5. Immunofluorescence Analysis and ER Staining

EA.hy926 (4 × 10^5^) or Jurkat (10^6^) cells were cultured in 6-well plates in growth medium as described above. After stress induction, cells were washed with PBS/0.5% FBS, fixed in 0.01% paraformaldehyde for 10 min, and permeabilized with 10% Tween-20/1% BSA for 10 min. EA.hy926 and Jurkat cells were incubated overnight with 1:100 anti-BiP rabbit polyclonal antibody (ab21685) at 4 °C. The cells were washed in PBS/0.5% FBS and stained with 1:100 fluorescein-labeled goat anti-rabbit IgG (02-15-06) for 40 min at room temperature. Data were acquired with a FACSCalibur (Becton-Dickinson Biosciences, San Jose, CA, USA) and analyzed with FCS Express v. 4.0 analysis software (De Novo Software, Glendale, CA, USA).

For live-cell imaging of the endoplasmic reticulum (ER), cells were incubated with either 100 nM DiOC_6_(3) (3,3′-dihexyloxacarbocyanine iodide; Thermo Fisher Scientific, Waltham, MA, USA) or 200 nM LumiTracker^®^ ER Green (Lumiprobe, Hannover, Germany) in serum-free medium for 20 min at 37 °C. Following incubation, the cells were washed twice with warm PBS to remove excess dye. Cells were then immediately visualized using a Nikon TE2000-C1 confocal microscope (Nikon, Japan) at excitation/emission wavelengths of 488/501 nm, or analyzed using a FACS Celesta flow cytometer equipped with FACSDiva software (version 8.0.1.1; BD Biosciences, San Jose, CA, USA).

### 2.6. Bioinformatic Analysis

Bioinformatic analysis was performed using the publicly available RNA-sequencing datasets GSE176365 (SRP323222) [34] and E-MTAB-2770 [35]. To enable cross-dataset comparison, FPKM values were converted to TPM (Transcripts Per Million), and a median ratio transformation was applied to align the expression scales between the datasets. Subsequently, genes were ranked based on the calculated TPM differences. The ranked gene list was subjected to gene set enrichment analysis (GSEA) [36,37] using a local installation of GSEA software (version 4.1.0; Broad Institute, Cambridge, MA, USA). Dataset-independent GseaPreranked mode was utilized against the c5.go.cc.v2026.1.Hs.symbols.gmt database. For the enrichment analysis, a false discovery rate (FDR) cutoff of <0.25 was applied, in accordance with the Broad Institute GSEA guidelines for exploratory data analysis.

### 2.7. RNA Preparation and Reverse-Transcription Quantitative Polymerase Chain Reaction (qRT-PCR)

Total RNA was isolated from Jurkat, EA.hy926, and FRSN cells using an RNeasy Mini Kit (Qiagen, Hilden, Germany) followed by DNase I treatment (Thermo Fisher Scientific, Waltham, MA, USA) according to the manufacturer’s protocols. One microgram of each RNA sample was reverse-transcribed using random hexamer primers with a RevertAid H Minus First Strand cDNA Synthesis Kit (Thermo Fisher Scientific, Waltham, MA, USA) following the manufacturer’s instructions. The resulting cDNA was used for qRT-PCR, which was performed using a CFX96Real-Time PCR System (Bio-Rad Laboratories, Hercules, CA, USA) and a Maxima SYBR Green/ROX qPCR Master Mix (Thermo Fisher Scientific, Waltham, MA, USA). Each reaction was run in triplicate. Amplification was performed for 10 min at 95 °C, followed by 40 cycles of 95 °C for 15 s, 55 °C for 30 s, and 72 °C for 30 s. Relative transcript expression was normalized to the GAPDH reference internal control mRNA from each sample using the 2^−ΔΔCt^ method. The primers for BiP, XBP1(S), XBP1(tot), and GAPDH were described previously [38].

### 2.8. Mathematical Modeling

Mathematical modeling was performed using CellDesigner software (version 4.4.2; The Systems Biology Institute, Tokyo, Japan). The *IRE1-XBP1* and *ATF6* signaling pathways were constructed through visual programming by incorporating corresponding biological entities (such as genes, mRNA, and proteins) into the model scheme. All kinetic interactions within the pathways were mathematically defined using the law of mass action.

### 2.9. Topography and Young’s Modulus Scanning

Scanning was performed using an ICAPPIC scanning ion-conductance microscope (ICAPPIC Ltd., London, UK). Nanocapillaries with a radius of 18–35 nm, fabricated using a P-2000 laser puller (Sutter Instrument, Novato, CA, USA), were used for all experiments. The approach velocity (loading rate) was set to 120 µm/s. Topographic imaging was conducted using non-contact mode with an ion current setpoint reduction of 0.5% from the baseline value. To calculate the Young’s modulus, surface indentation (deformation) was performed until the current dropped by 1% and 2%. All measurements were carried out in Hank’s Balanced Salt Solution (HBSS, PanEco, Moscow, Russia).

### 2.10. Statistical Analysis and Temporal Analysis of Gene Expression

Data were collected from at least three independent experiments with three replicates each. To identify time points where mRNA levels differed significantly from controls, the Mann–Whitney U test was applied to experimental and control ΔCt values. The temporal dynamics of mRNA and protein levels were modeled using a three-parameter exponential function (y = exp(a + b×x + c×x^2^)) and a quartic polynomial function, respectively. Numerical simulations and nonlinear curve fitting were performed in OriginPro (OriginLab Corporation, Northampton, MA, USA) using the Levenberg–Marquardt iteration algorithm. The response time (ResTime), defined as the time to reach maximum expression, was derived as a parameter calculated using the formula ResTime = −b/(2×c). Interpolated ResTime values and their standard errors were compared using Z-statistics. The percentage of spliced XBP1 mRNA was calculated as the ratio of 2^−ΔCt^ XBP1(S) to 2^−ΔCt^ XBP1(tot). The magnitude of differences between the groups was quantified using Cohen’s effect size *d* along with the traditional Student’s *t*-test. The effect sizes *d* were interpreted as small (0.2), medium (0.5), and large (0.8) according to Cohen’s conventions [39].

## 3. Results

### 3.1. Morphofunctional Variations in the ER Compartment

To analyze the cellular stress responses, we proceeded from the proposition that the expression of the stress-responsive genes exhibits impulse-like characteristics [40,41,42], i.e., it is possible to detect pronounced maximal gene expression at a certain time interval after stress induction. Specifically, we took the response time for the mRNA of a given gene to be the time required for expression to reach its mRNA maximum. We used cell lines with high and low ER volumes: human endothelial EA.hy926 cells and Jurkat T-cells.

**Figure 1:**
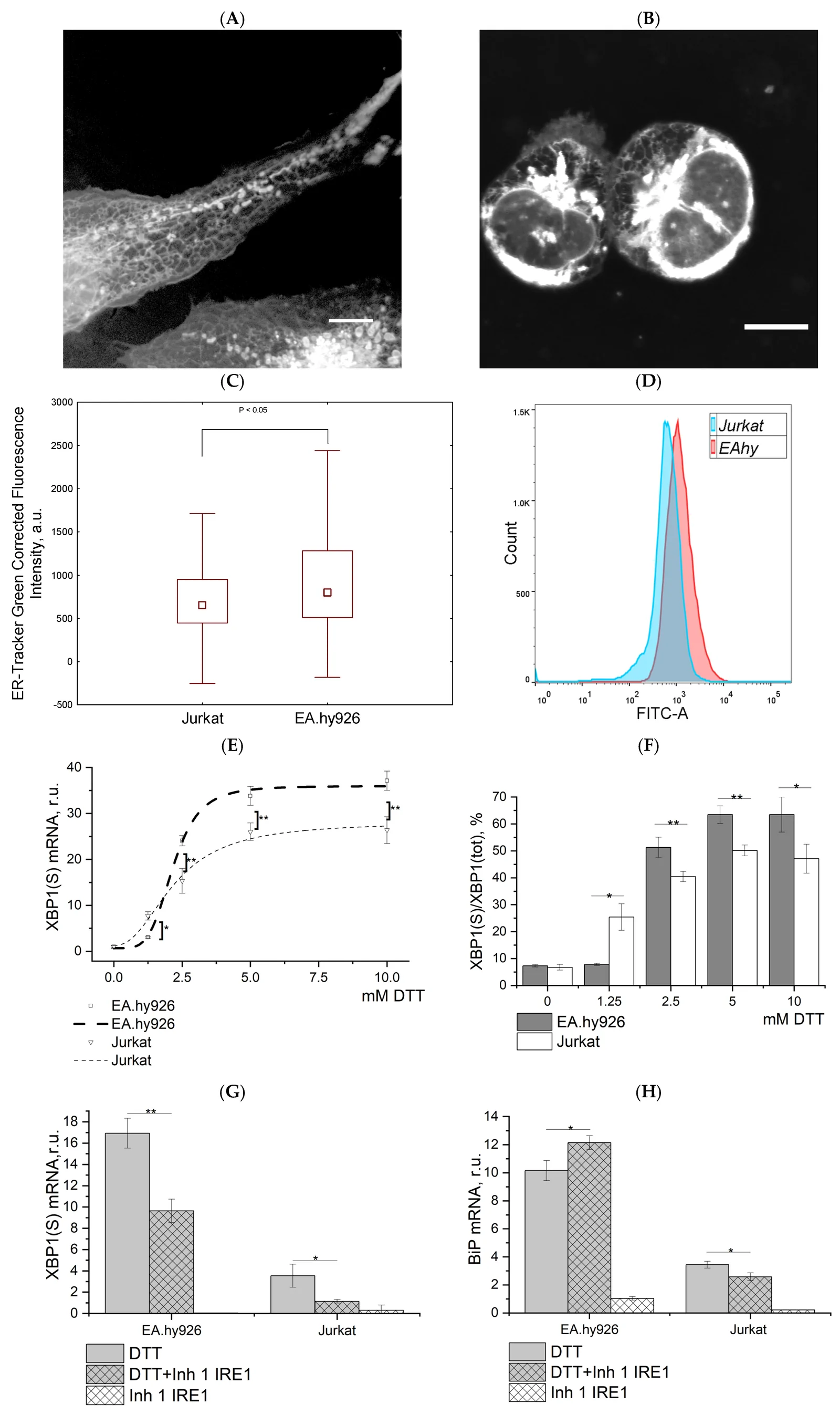
IRE1α−XBP1 signaling pathway in Jurkat and EA.hy926 cells. (**A**,**B**) Confocal micrographs of EA.hy926 (**A**) and Jurkat (**B**) cells stained with the fluorescent probe DiOC6(3) (live imaging). Scale bar: 5 µm. (**C**) Mean fluorescence intensity (MFI) of EA.hy926 and Jurkat cells stained with ER-Tracker Green (LumiTracker^®^ ER Green), corrected for the autofluorescence of unstained cells (see text for details). (**D**) Representative histograms of EA.hy926 and Jurkat cells stained with ER-Tracker Green. (**E**,**F**) Dose–response of the *IRE1α-XBP1* signaling pathway for *XBP1* mRNA splicing: Jurkat and EA.hy926 cells were treated with 1.25–10 mM DTT for 4 h, followed by total RNA isolation and cDNA synthesis. qPCR was performed using transcript variant-specific primers to simultaneously detect both spliced and total pools of *XBP1* mRNAs: (**E**) *XBP1(S)* mRNA expression in Jurkat and EA.hy926 cells; (**F**) the *XBP1(S)/XBP1(tot)* mRNA ratio in Jurkat and EA.hy926 cells. The data reveal that *IRE1α-XBP1* signaling in Jurkat cells is sensitive to low DTT concentrations, whereas it is insensitive in EA.hy926 cells, while both cell lines plateau at concentrations greater than or equal to 5.0 mM. (**G**,**H**) The effects of IRE1α inhibition on *XBP1(S)* and *BiP* mRNA levels during ER stress. *XBP1(S)* (**G**) and *BiP* (**H**) mRNA levels with and without IRE1α inhibition in EA.hy926 and Jurkat cells treated with 2.5 mM DTT for the indicated times. The data confirm the strong dependence of *BiP* levels on *IRE1α-XBP1* signaling in Jurkat cells and possible overcompensation of *IRE1α-XBP1* signaling inhibition in EA.hy926 cells. ** *p* < 0.01; * *p* < 0.05. Data are representative of three independent experiments.

Confocal imaging of the ER network using DiOC_6_ (Figure 1A,B) revealed that both cell lines exhibited a continuous and well-defined reticular network with a striking difference in the ER volumes: while EA.hy926 cells possess an expansive, spread-out ER characteristic of active secretory cells, Jurkat cells are characterized by a minimal cytoplasmic rim where the ER is confined to a narrow perinuclear space. Flow cytometry utilizing the LumiTracker^®^ ER Green probe (Lumiprobe, Hannover, Germany) demonstrated differences between the two cell lines in fluorescence intensity, corrected for the autofluorescence of unstained cells (Figure 1C,D), revealing a larger ER volume in EA.hy926 cells compared to Jurkat cells.

To further confirm the differences in the development of the secretory pathway between the two cell types, we performed a bioinformatic analysis using publicly available RNA-sequencing datasets. The GSE176365 (SRP323222) [34] dataset was used for the EA.hy926 line, and E-MTAB-2770 [35] was utilized for the Jurkat line. Following normalization, gene set enrichment analysis (GSEA) was conducted. In EA.hy926 cells, GSEA revealed the enrichment of Gene Ontology Cellular Component (GO:CC) gene sets, including rough endoplasmic reticulum (FDR = 0.22), endoplasmic reticulum Golgi intermediate compartment (FDR = 0.22), smooth endoplasmic reticulum (FDR = 0.23), and endoplasmic reticulum lumen (FDR = 0.23), indicating an elevated abundance of transcripts involved in maintaining an expanded ER network (see Supplementary Table S1). These highly expressed genes in EA.hy926 cells, along with others such as Golgi lumen (FDR = 0.18) and vesicle lumen (FDR = 0.20), collectively reflect a more robustly developed secretory pathway in EA.hy926 cells compared to Jurkat cells. Notably, EA.hy926 cells expectedly showed an enrichment of gene sets characteristic of adherent cells—including those related to the cytoskeleton, cell–cell junctions, and cell–matrix interactions—as well as gene sets associated with active endocytosis, a hallmark of endothelial cells. When comparing the expression levels of individual key genes, we observed 6-fold higher expression of *SEC61A1*, a core translocon component; a 56-fold increase in *CKAP4* (CLIMP-63), which anchors the ER to microtubules; and an 11-fold elevation in *HSPA5* (BiP), the primary ER chaperone. Taken together, these findings demonstrate a substantially greater protein-synthesizing and folding capacity of the ER in EA.hy926 cells. Interestingly, differences were also observed in the expression of structural proteins responsible for ER architecture. Specifically, we noted divergent expression of reticulons, which stabilize ER membrane curvature: *RTN2* expression was upregulated 39-fold, which may reflect active secretory traffic, while *RTN4* showed a 2.4-fold increase, suggesting a moderate expansion of the tubular ER in EA.hy926 cells.

Distinct differences were also observed between EA.hy926 and Jurkat cells in the expression levels of the UPR signaling branches. In EA.hy926 cells, *ATF6* expression was 1.6-fold higher, concurrently with its target genes *SEL1L* (an ERAD-associated protein component) and *HERPUD1*, which showed 1.3-fold and 2-fold increases, respectively. Furthermore, *ERN1* (encoding IRE1) expression was 2.2-fold higher in EA.hy926 cells. Concurrently, it is worth noting that *EIF2AK3* (encoding PERK) mRNA expression was 2-fold lower in these cells. Analysis of the E-MTAB-9372 dataset [43] revealed that among fetal endothelial cells from the gut, lungs, and kidneys, only HUVECs exhibited reduced PERK expression. This downregulation may reflect a hypoxic adaptation inherited from the parental HUVEC line. Developing under conditions of prenatal physiological hypoxia, the umbilical endothelium possibly suppresses PERK levels to prevent premature and destructive attenuation of global translation at low pO_2_, thereby preserving endothelial function—particularly its antithrombotic properties—and maintaining fetal perfusion. Notably, elevated *ATF6* levels were restricted exclusively to HUVECs and lung endothelial cells.

In contrast, GSEA for Jurkat cells highlighted a distinct transcriptional profile focused predominantly on mitochondrial bioenergetics and cell cycle progression machinery (Supplementary Table S2). Specifically, prominent enrichment was observed for gene sets associated with mitochondrial respiration, including the NADH dehydrogenase complex, the proton-transporting ATP synthase complex, and the respiratory chain complex. Additionally, Jurkat cells demonstrated marked enrichment of structural components governing DNA duplication and chromosome segregation, such as the replisome, the nuclear replication fork, the DNA polymerase complex, and kinetochore components. Collectively, these bioinformatic data corroborate the miniature, low-secretory morphology of Jurkat T-lymphoblasts. This indicates that their metabolic resources are fundamentally allocated toward rapid proliferation and mitochondrial energy production rather than the maintenance of an expanded endoplasmic reticulum network and active protein trafficking, which characterizes the secretory phenotype of EA.hy926 cells.

### 3.2. Dose Response of IRE1α-XBP1 Signaling

To induce ER stress in Jurkat and EA.hy926 cells, we used dithiothreitol (DTT), which disrupts disulfide bonding and causes the accumulation of misfolded proteins in the ER lumen directly [44]. A number of processes directly associated with ER stress were previously observed among the most significant upregulated gene sets in Jurkat cells treated with DTT [45].

To find the optimal concentration of the ER stress inducer (DTT) in Jurkat and EA.hy926 cell lines at which IRE1α-XBP1 signaling would be activated significantly but not saturated, we performed a dose–response analysis of the IRE1α-mediated splicing of XBP1 mRNA via assessment of the baseline-normalized levels of spliced (XBP1(S)) and unspliced (XBP1(U)) XBP1 mRNAs in cells treated with different concentrations of DTT for 4 h. We observed a monotonic increase in the relative XBP1(S) level with respect to DTT concentration in both cell lines, and this dependence is well approximated by a logarithmic function (Figure 1E). The initial relative increase in spliced XBP1 (XBP1(S)) mRNA levels after a 4 h exposure to 1.25 mM DTT was more pronounced in Jurkat cells (approx. 8.0-fold) than in EA.hy926 cells (2.0-fold). This result appears to indicate the depletion of the BiP pool and IRE1α activation in Jurkat cells, whereas EA.hy926 cells were characterized by a higher sensitivity threshold. Sharp activation of IRE1α-XBP1 signaling occurred at 2.5 mM DTT in EA.hy926 cells, but the normalized amplitude of XBP1 mRNA splicing upregulation was similar to that in Jurkat cells treated with 2.5 mM DTT (Figure 1F). XBP1(S) mRNA levels plateaued at 5–10 mM DTT in both cell lines (Figure 1D). An assessment of the dynamic range of IRE1α-XBP1 signaling based on approximated dose–response curves yielded a similar four-fold dynamic range for both cell lines.

Based on the dose–response analysis, we selected a 2.5 mM DTT concentration for subsequent comparisons, which corresponds to a non-saturating signaling level.

### 3.3. The Effect of Small-Molecule Inhibition of IRE1α Endoribonuclease on BiP Expression

To clarify the extent to which the expression of BiP upon ER stress depends on IRE1α-XBP1 signaling, we tested whether specific inhibition of IRE1α endoribonuclease activity has the same effect on BiP mRNA and protein expression in Jurkat and EA.hy926 cells. To selectively inhibit the endoribonuclease domain without affecting the kinase function of IRE1α, we used STF 083010, a cell-permeable small molecule that covalently binds to the active center of the IRE1α endoribonuclease, blocking its activity [46].

Treatment with STF 083010 inhibited IRE1α-mediated XBP1u mRNA splicing by 50% in EA.hy926 cells (*p* = 0.029) and by 68% in Jurkat cells (*p* < 0.05) (Figure 1G). In Jurkat cells, IRE1α inhibition led to a significant decrease in BiP mRNA levels (*p* < 0.01), confirming that BiP expression is dependent on IRE1α-XBP1 signaling in this line (Figure 1H). Conversely, an unexpected, statistically significant increase in BiP mRNA was observed in EA.hy926 cells upon inhibition (*p* = 0.048). This suggests that, while IRE1α-XBP1 signaling may play a minor role in regulating BiP in EA.hy926 cells, compensatory upregulation via IRE1α-independent pathways may occur. Although the exact mechanism remains unclear, the potential involvement of RIDD suggests a reciprocal IRE1α-BiP relationship, where IRE1α endoribonuclease activity and independent pathways exert competing effects on BiP expression. These findings indicate that the IRE1α-XBP1 arm of the UPR contributes differentially to BiP regulation across different cell types.

### 3.4. Analysis of Temporal Patterns in ER Stress-Associated Gene Expression

**Figure 2:**
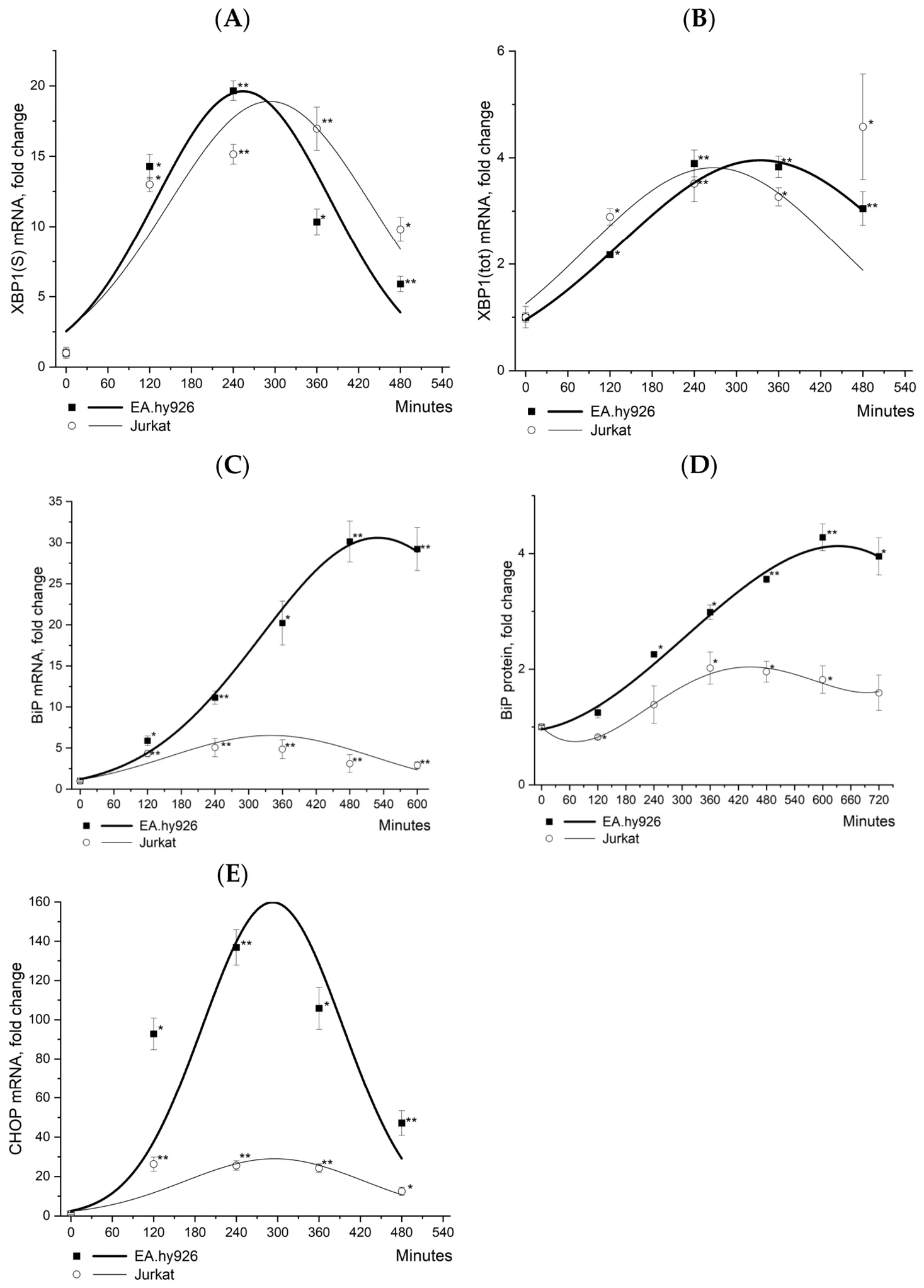
Temporal profiles of UPR gene expression in EA.hy926 vs. Jurkat cells. Jurkat and EA.hy926 cells were treated with 2.5 mM DTT for the indicated times, followed by total RNA isolation, cDNA synthesis, and qPCR utilizing transcript variant-specific primers. (**A**,**B**) *XBP1(S)* mRNA levels (**A**) and *XBP1(tot)* mRNA levels (**B**). The data reveal similar temporal profiles of *XBP1(S)* in Jurkat cells compared to EA.hy926 cells, but differences in *XBP1(tot)* upregulation at the 8 h time point. (**C**,**D**) *BiP* mRNA and protein levels. The data indicate distinct response times for *BiP* mRNA (**C**) and BiP protein (**D**) expression between Jurkat and EA.hy926 cells. (**E**) *CHOP* mRNA levels. The data reveal similar temporal profiles but dramatically different magnitudes of *CHOP* mRNA expression between Jurkat and EA.hy926 cells. * *p* < 0.05 and ** *p* < 0.01 (stressed vs. control cells). Data are representative of three independent experiments.

To determine the response times for XBP1s, BiP, and CHOP, we measured their temporal expression profiles in each cell line using a fixed concentration of DTT (2.5 mM) (Figure 2). The resulting profiles were fitted using an exponential function. The XBP1s mRNA response time was 254 ± 25 min (mean ± SE) for EA.hy926 cells and 292 ± 42 min for Jurkat cells; no significant difference was observed between the lines (z = 0.77). In both cell types, XBP1s mRNA reached peak levels at approximately the same time, followed by a gradual decline (Figure 2A). Additionally, a 50% attenuation of IRE1α-XBP1 signaling occurred at 434 min in EA.hy926 cells and 458 min in Jurkat cells, which was not a statistically significant difference. The maximum expression levels, expressed as fold changes, were nearly identical in both lines.

We also analyzed the total XBP1 mRNA pool (XBP1tot), comprising both the XBP1u substrate and the spliced XBP1s form (Figure 2B). The XBP1tot mRNA response time was 334 min with SE = 16 min for EA.hy926 cells and 267 with SE = 30 min for Jurkat cells, representing a significant difference (z = 1.98). Despite the temporal differences, the overall magnitude of XBP1tot upregulation was similar in both cell types.

BiP has been reported to affect IRE1α attenuation [18]. Thus, we compared the dynamics of IRE1α-XBP1 signaling with BiP mRNA response times. The BiP mRNA response time was 340 min for Jurkat cells (SE = 29 min) versus 530 min for EA.hy926 cells (SE = 38 min), a statistically significant difference (z = 3.93) (Figure 2C). Notably, the response amplitude differed substantially, reaching 30-fold in EA.hy926 cells compared to 5-fold in Jurkat cells. These BiP mRNA response times correlate with the basal ER folding capacity of the respective cell lines. The faster response for BiP mRNA and protein in Jurkat cells was accompanied by a similar time of attenuation for IRE1α-XBP1 signaling as that of EA.hy926 cells.

In general, mRNA changes corresponded to protein levels (Figure 2D). The BiP protein response time in Jurkat cells of 494 min (SE = 22 min) was 1.3 times faster with a lower amplitude than in EA.hy926 cells, with a response time of 632 min (SE = 79 min). Both cell lines exhibited an initial lag phase in protein levels, likely reflecting the depletion of the free chaperone pool due to its sequestration by non-native proteins. This lag was more pronounced in Jurkat cells, suggesting a significantly smaller initial pool of free chaperones. While BiP facilitates the processing of defective proteins during ER-associated degradation (ERAD), its own degradation or loss via aggregation is unlikely to be substantial. Alternatively, this decline may be attributed to ER-phagy, which can directly reduce chaperone levels.

The temporal patterns of BiP and XBP1s expression were more closely correlated in Jurkat cells. In EA.hy926 cells, the decline in XBP1s levels during prolonged stress (up to 480 min) did not lead to a significant reduction in BiP mRNA. This may reflect a lower reliance of BiP expression on XBP1-mediated regulation in EA.hy926 cells and a potentially greater contribution of the ATF6 pathway.

Given that the transcription factor CHOP (CCAAT/enhancer-binding protein homologous protein) is positively regulated by ATF6 and the PERK-ATF4 branches of the UPR [31,47,48], we compared CHOP mRNA expression in EA.hy926 and Jurkat cells. The dynamics of CHOP mRNA expression in Jurkat cells were similar to those in endothelial cells, with no statistically significant difference in response time (292 min for EA.hy926 cells (SE = 33 min) and 295 min for Jurkat cells (SE = 33 min)), but a significantly larger amplitude in EA.hy926 cells (Figure 2E).

### 3.5. Modeling of Temporal Patterns in ER Stress-Associated Gene Expression

One of the factors that may explain the observed differences in the temporal expression profiles of UPR-induced genes is the volume of the ER compartment. To evaluate this, we constructed a mathematical model of the IRE1 and ATF6 branches of the UPR for cells with small and large ER compartments. In the model, we established a five-fold difference in the ER volume ratio between ‘small-ER’ cells (Jurkat) and ‘large-ER’ cells (EA.hy926). In typical lymphocytes, the cytoplasmic volume is minimal and the ER may occupy only 1–3% of the cell volume, whereas in active endothelial cells, this fraction can reach up to 10–15% or higher, particularly in zones of active angiogenesis. Thus, our established difference represents a conservative estimate. Our observations using DiOC6(3) staining (Figure 1A,B) as well as flow cytometry data with ER-Tracker Green (Figure 1C,D) and differences in transcriptomes (Supplementary Tables S1 and S2) corroborate the difference in ER volume used in our numerical simulations.

The levels of constitutively expressed proteins correlate with cell size, as determined by parameters such as forward and side scatter in flow cytometry, carboxyfluorescein succinimidyl ester (CFSE) total protein staining, nuclear volume, and dry mass measured via quantitative phase microscopy (QPM) [49]. Accordingly, most proteins and RNAs maintain constant concentrations during cell growth [50], and organelle volumes typically scale proportionally with cell size. However, organelle-to-cell volume ratios can vary significantly across different cell types [51]. Given that transcription and translation rates are proportional to cell size [52], and consistent with the observed ER volume differences, we assigned proportional initial amounts of BiP protein, mRNA, and other UPR components in our model.

**Figure 3:**
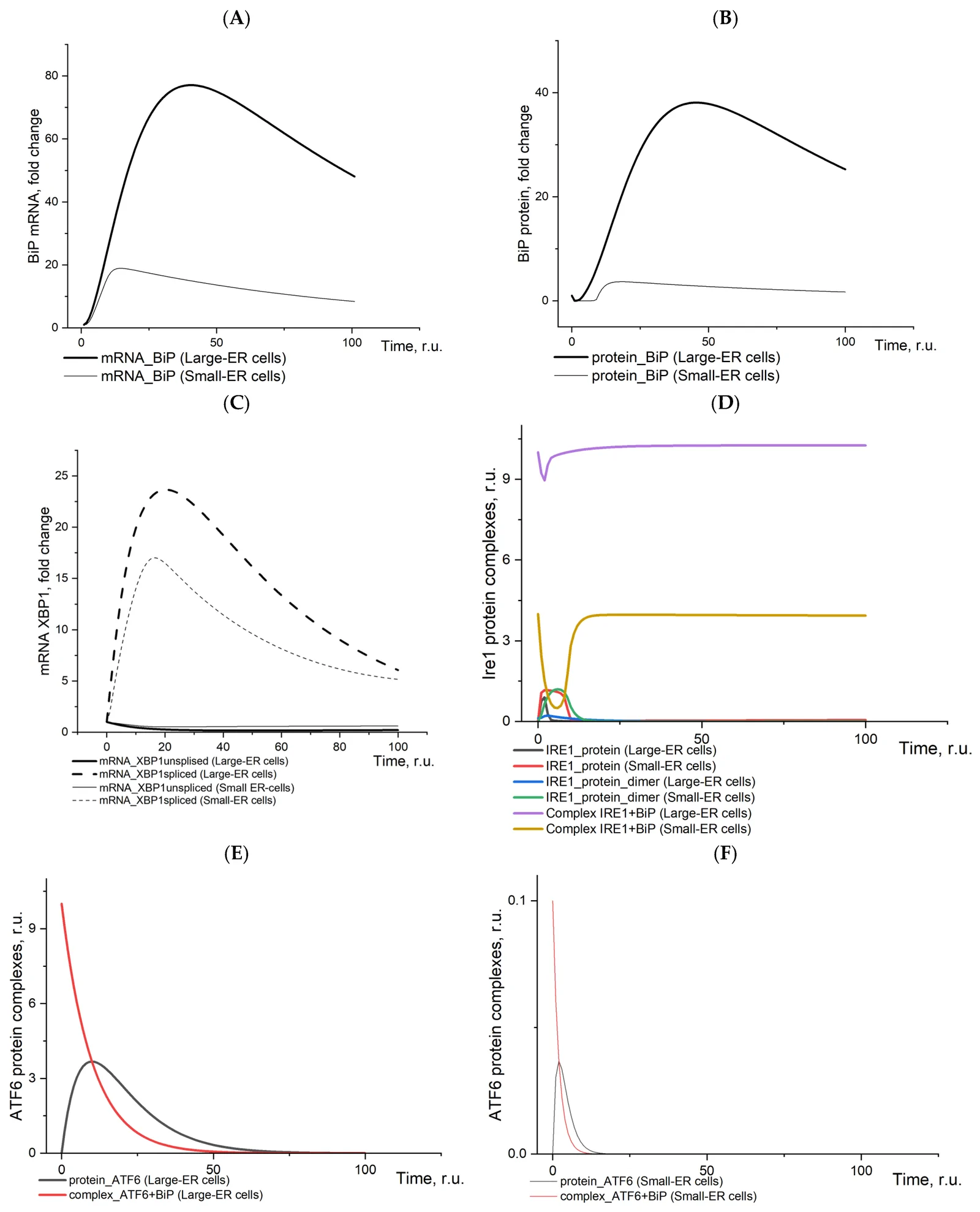
The modeled temporal profiles of UPR genes in ‘small-ER’ (Jurkat) and ‘large-ER’ (EA.hy926) cells. CellDesigner was used for model construction. (**A**,**B**) *BiP* mRNA (**A**) and BiP protein (**B**) levels, indicating distinct response times between the two cell lines. (**C**) *XBP1(S)* and total *XBP1* mRNA levels, revealing similar temporal profiles of *XBP1(S)* across both lines. (**D**) The dynamic distribution of IRE1 species. (**E**,**F**) The temporal profiles of ATF6 signaling components.

A robust and identical level of ER stress was applied to both lines, defined by an initial concentration of unfolded proteins exceeding the concentration of free BiP. As shown in Figure 3A, the BiP response times were less in ‘small-ER’ cells (Jurkat): 2.93 times for mRNA and 2.42 for protein. The model with current parameters overestimates the experimentally observed differences 1.56 and 1.3 times but demonstrates a similar relation between mRNA and protein response times. Notably, the model replicates the lag phase in free BiP levels, which is more pronounced in ‘small-ER’ cells (Jurkat), where levels are initially below baseline. The decline in free BiP in this context is primarily driven by the formation of complexes with unfolded proteins.

The more pronounced decline in free BiP levels in ‘small-ER’ cells leads to a delayed re-association of the IRE1–BiP complex (Figure 3D) and persistence of IRE1 dimers compared to ‘large-ER’ cells. However, this does not significantly alter the dynamics of spliced XBP1 levels: the response time was 1.25 times less in ‘small-ER’ cells (Figure 3C). A limitation of our model is the exclusion of ATF6 gene transcription, which results in the depletion of the initial ATF6 protein pool. The amplitudes of the ATF6 transcription factor levels differ substantially between the two cell types (Figure 3E,F). Nevertheless, the model successfully confirms the differences in BiP response times observed across both cell lines.

### 3.6. Analysis of Temporal Patterns in ER Stress-Associated Gene Expression in Senescent Fibroblasts

Despite the conserved, non-specific nature of the UPR across various cell types, its signaling dynamics—specifically the BiP response time—differ significantly between EA.hy926 and Jurkat cells. Our mathematical model suggests that the size of the ER compartment is a critical factor driving these differences. Notably, an increase in overall cell size is a hallmark of cellular senescence and has been shown to potentially induce the senescent phenotype [50]. Given that senescence is characterized by a decline in proteostasis, we propose that cell enlargement, alongside the biochemical shifts, further modulates UPR signaling dynamics.

**Figure 4:**
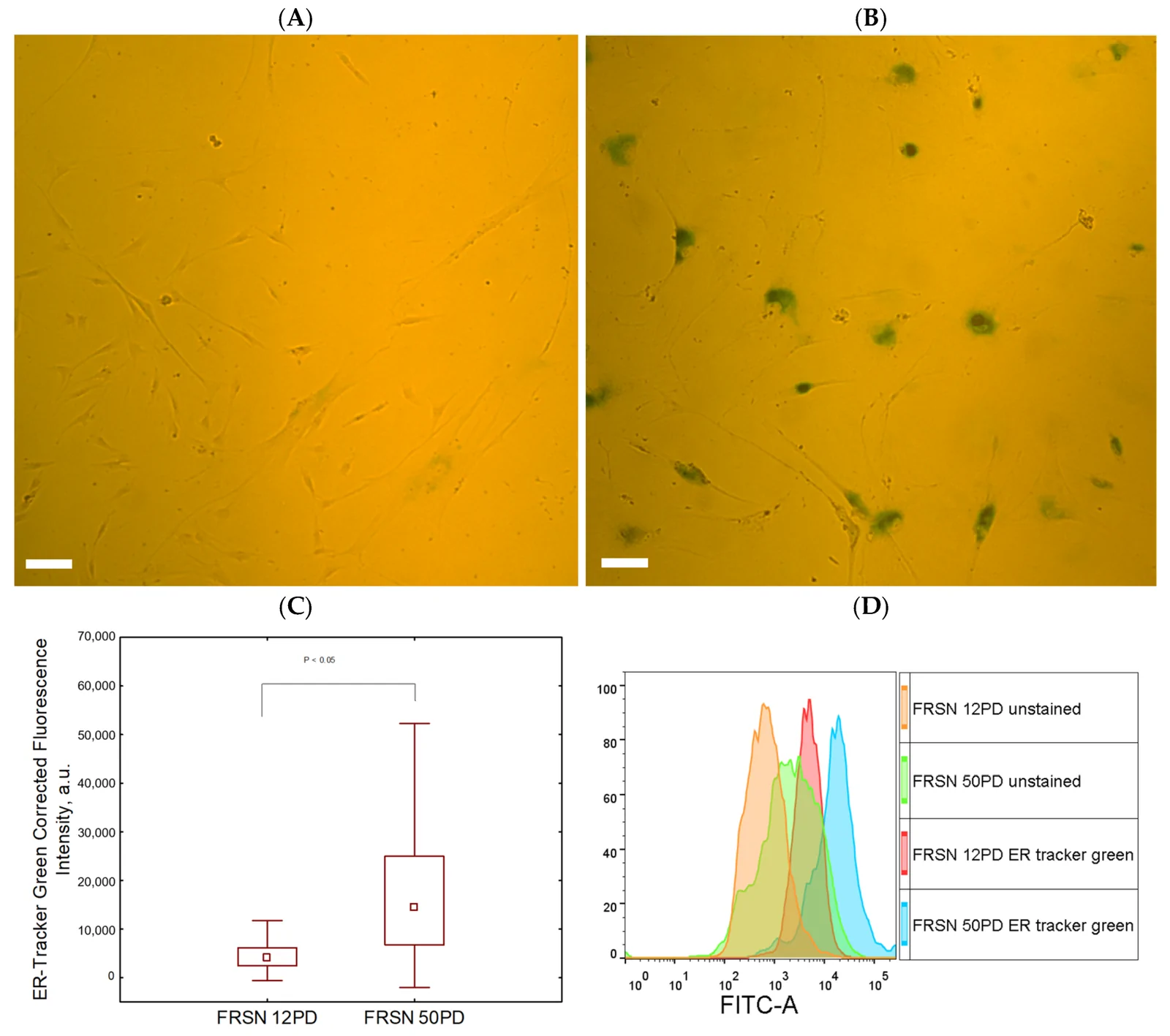

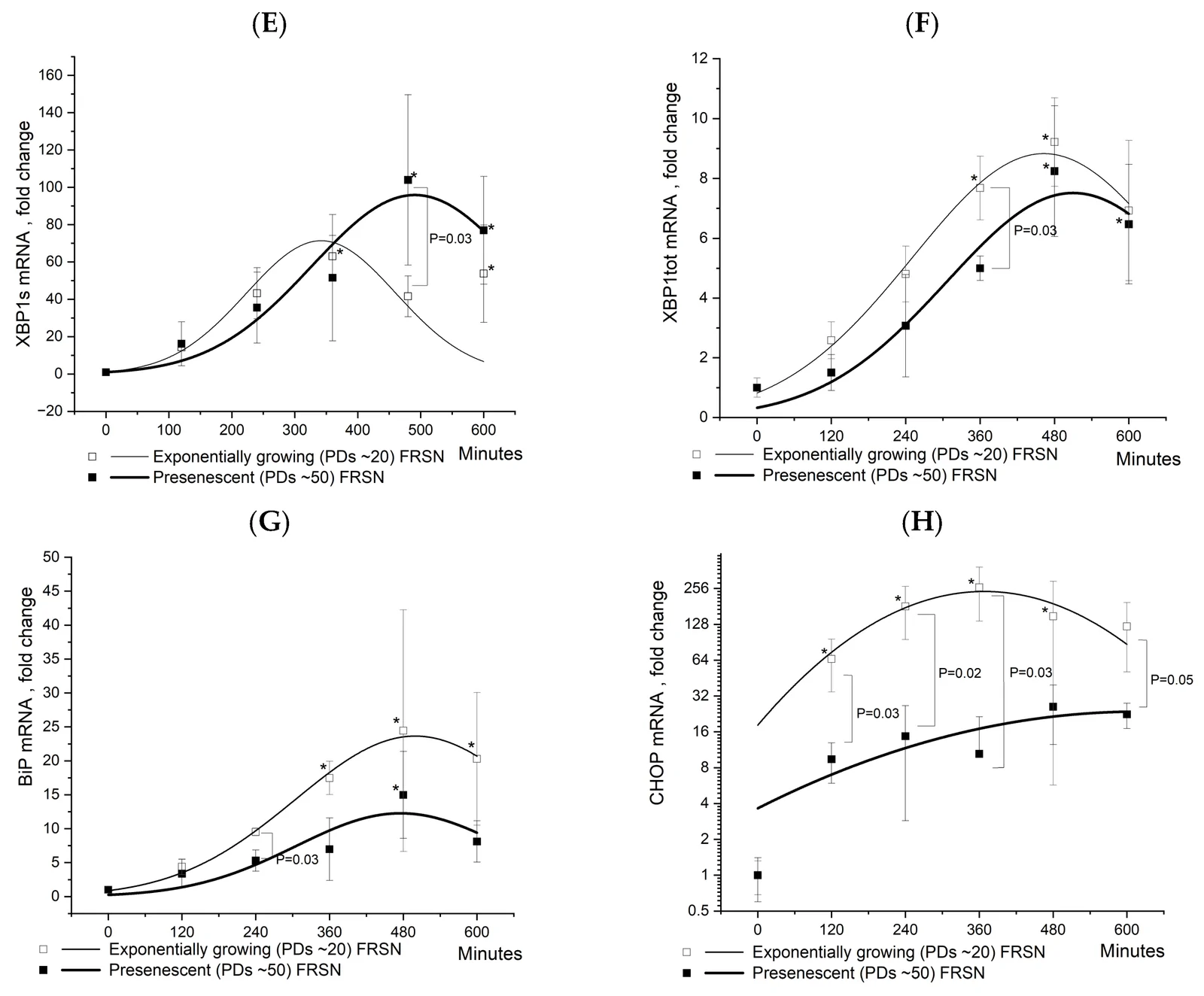
The temporal profiles of UPR gene expression in FRSN cells during replicative senescence. (**A**,**B**) Senescence-associated beta-galactosidase (SA-β-gal) activity analysis. SA-β-gal activity was analyzed in exponentially growing FRSN cells at the 12th population doubling (**A**) and presenescent cells at the 50th population doubling (**B**) using X-gal substrate. Late-passage fibroblasts exhibit high levels of SA-β-gal activity, characteristic of the replicative senescence phenotype. Scale bar: 100 µm. (**C**) The mean fluorescence intensity (MFI) of FRSN cells at the 12th and 50th population doublings stained with ER-Tracker Green (LumiTracker^®^ ER Green), corrected for the autofluorescence of unstained cells (see text for details). (**D**) Representative histograms of FRSN cells at the 12th and 50th population doublings unstained and stained with ER-Tracker Green. (**E**,**F**) *XBP1(S)* (**E**) and total *XBP1* (**F**) mRNA levels. The data reveal a protracted temporal profile of *XBP1(S)* in presenescent FRSN cells. (**G**) *BiP* mRNA levels. The data indicate similar *BiP* mRNA response times in presenescent and exponentially growing FRSN cells, but a lower magnitude and an extended lag phase in senescent cells. (**H**) *CHOP* mRNA levels. The data reveal similar temporal profiles but a significantly lower magnitude of *CHOP* expression in presenescent FRSN cells. * *p* < 0.05 (stressed vs. control cells). Data are representative of three independent experiments.

Senescent cells can adapt to adverse environments by mobilizing cellular stress responses, which play a critical role in systemic aging and age-related pathologies. Whether the cell adaptive capacity and the specific dynamics of their stress responses are altered in senescent cells remains poorly understood. To address this, we employed a model of replicative senescence using FRSN fibroblast-like cells to evaluate the response times of key UPR genes. After long-term cultivation under standard conditions, cells at late passages (corresponding to 50 population doublings) exhibited characteristic morphological shifts, including cell enlargement and high beta-galactosidase activity (Figure 4B) [42]. As previously reported [53], gene expression analysis in these presenescent cells compared with exponentially growing controls (Figure 4A) confirmed significant alterations in markers of cellular senescence.

Flow cytometric analysis of exponentially growing FRSN cells (12th PD) compared to presenescent cells (50th PD) demonstrated an increase in cell size along with a significant elevation in autofluorescence during replicative senescence (Figure 4D). Staining cells with ER-Tracker Green followed by autofluorescence correction revealed that presenescent cells were characterized by substantial expansion of the ER compartment (Figure 4C).

The XBP1s mRNA response time was 342 ± 22 min for exponentially growing FRSN cells and 491 ± 42 min for presenescent FRSN cells, representing a statistically significant difference (z = 3.63) (Figure 4E). Despite this temporal shift, differences in expression amplitude were minor. These results indicate that XBP1s-mediated stress signaling in presenescent cells is prolonged, characterized by a delayed peak. Analysis of the BiP expression time series showed a response time of 515 ± 24 min for exponentially growing cells versus 484 ± 45 min for presenescent cells, which was not a significant difference (z = 0.6) (Figure 4G). However, the increase in BiP mRNA levels in presenescent cells was characterized by a significantly lower amplitude and a longer lag phase compared to exponentially growing cells (6 h vs. 4 h) (Figure 4G). A significantly lower amplitude and an increased lag phase were also recorded for the XBP1tot expression profile, with response times of 479 ± 18 min for exponentially growing cells and 645 ± 127 min for presenescent cells (Figure 4F). Thus, presenescent cells exhibit an identical BiP mRNA response time but a substantially lower amplitude and a prolonged phase of initial unresponsiveness. This is coupled with a protracted XBP1s expression profile, suggesting a longer duration of IRE1 activity, potentially due to insufficient BiP levels. Concurrently, presenescent cells show a significantly lower CHOP expression amplitude (Figure 4H), which is consistent with the enhanced resistance of senescent cells to apoptosis.

### 3.7. Analysis of Cellular Stiffness in Senescent Fibroblasts

Alterations in UPR dynamics may be linked to the specific physical properties of senescent cells, which change due to an increase in cell size and a decline in proteostasis. To investigate this connection, we evaluated the stiffness of presenescent FRSN cells relative to exponentially growing controls.

**Figure 5:**
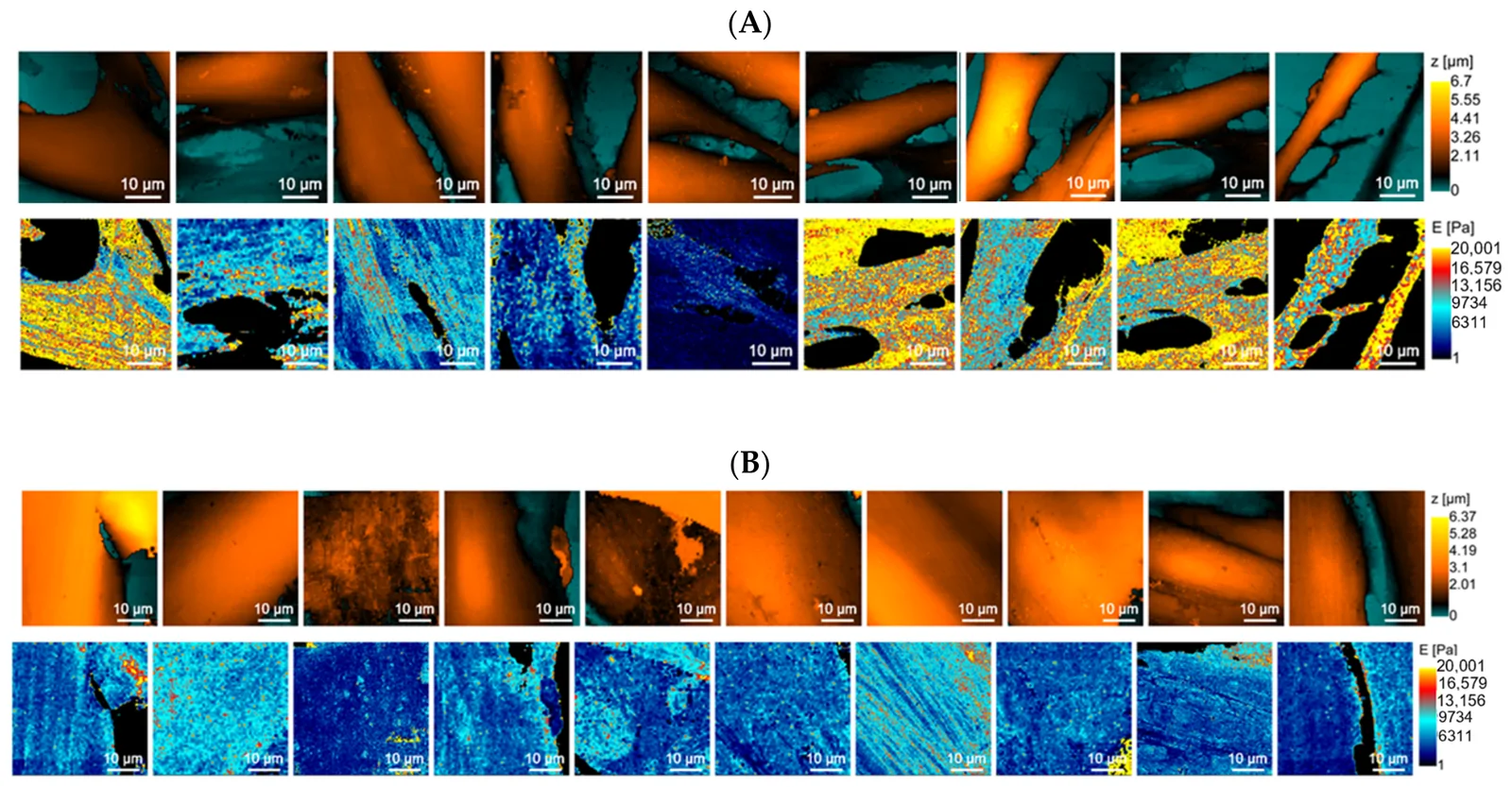

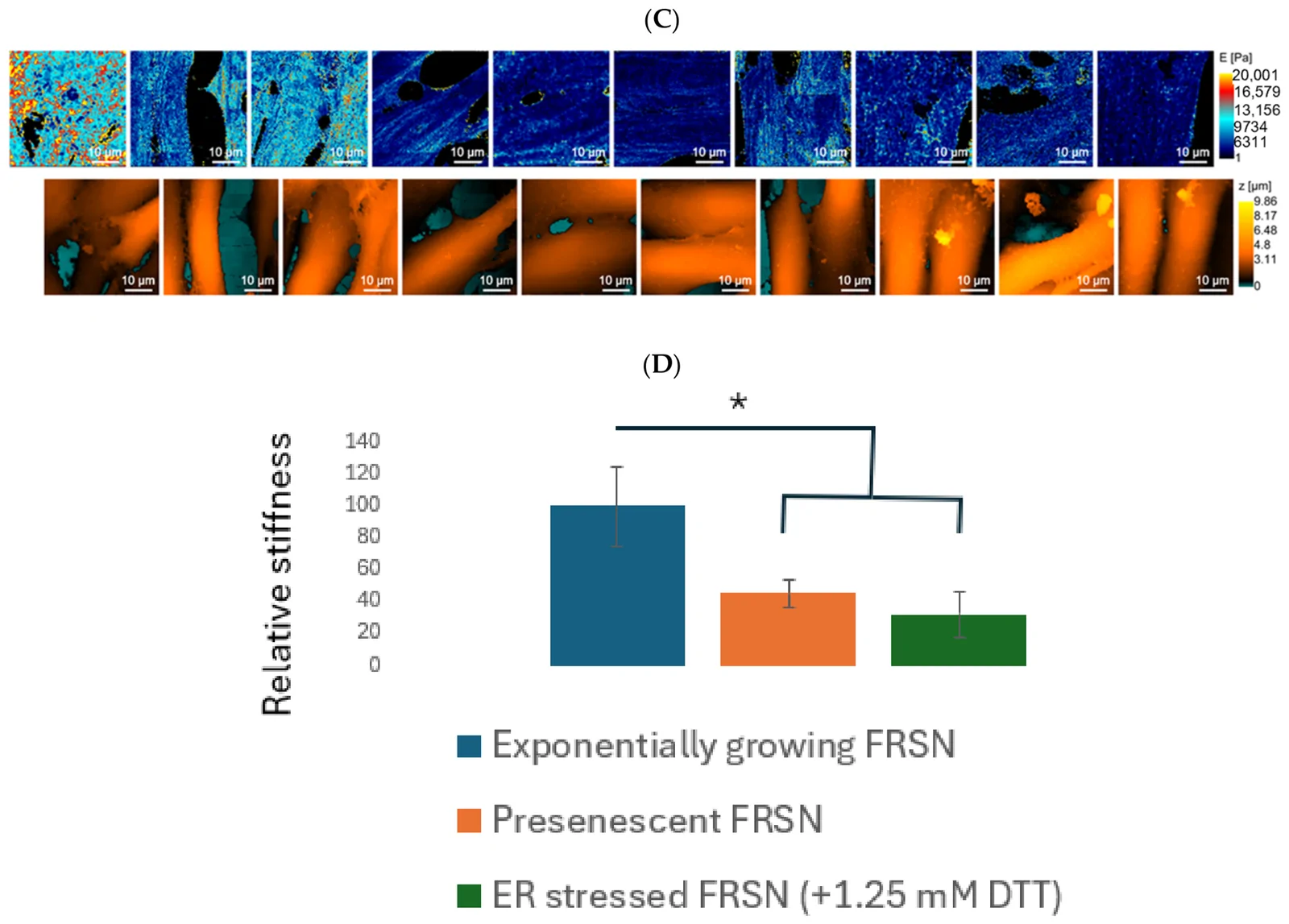
Scanning ion-conductance microscopy of presenescent FRSN cells. Topography and Young’s modulus of exponentially growing (**A**,**D**), presenescent (**B**,**D**), and ER-stressed (**C**,**D**) FRSN fibroblasts. The stiffness of the near-surface layer in presenescent and ER-stressed cells is significantly lower than that of exponentially growing cells. Data are representative of three independent experiments. * *p* < 0.05.

Using scanning ion-conductance microscopy (SICM), we compared the stiffness (Young’s modulus) of exponentially growing (Figure 5A,D), presenescent (Figure 5B,D), and ER-stressed FRSN fibroblasts. Additionally, we analyzed FRSN cells subjected to chronic ER stress (1.25 mM DTT for 24 h) (Figure 5C,D), a condition previously shown to induce a senescent phenotype similar to replicative senescence. In confluent FRSN cultures, the Young’s modulus was significantly higher in exponentially growing cells compared to both presenescent and stress-induced senescent cells, with the latter two groups exhibiting similar values. We hypothesize that the reduced stiffness in presenescent and stressed cells may be attributed to several factors. Given that SICM exerts less intense mechanical force on cells than atomic force microscopy (AFM) [54], changes in the near-surface layer—specifically the actin cortex—may significantly contribute to the observed effect. Due to the non-contact nature of SICM, which relies on ion current feedback rather than physical indentation, SICM is uniquely suited for probing the mechanical properties of the near-surface layer without triggering compensatory mechanical responses often observed in AFM. It appears that cellular senescence and ER stress may lead to the rarefaction and/or partial disorganization of the cytoskeleton. Furthermore, a reduction in cytoplasmic density may contribute to the observed softening. This aligns with the concept of macromolecular crowding reduction (cytoplasmic dilution) resulting from the scaling failure of biosynthesis relative to the disproportionate volume increase in senescent cells [55].

## 4. Discussion

In this study, we used the metric of gene response time to quantify UPR dynamics across cell types. This metric is specifically usable for induced genes exhibiting a pulsatile or transient response pattern, such as those involved in the cellular stress response. We defined the response time as the time required to reach the peak expression level (calculated from the fitted exponential models), providing an opportunity for comparative kinetic analysis. Qualitatively non-specific ER stress responses of the studied cell lines demonstrate specificity in the recovery kinetics of ER folding capacity. Specifically, Jurkat cells, which exhibit a low basal folding capacity, are characterized by shorter *BiP* mRNA and protein response times compared to EA.hy926 cells, which possess a higher ER volume. Our constructed mathematical model of the UPR, which compares cells with large and small ER compartments using an established five-fold difference in ER volumes, confirms a longer response time for cells with an expanded ER. At the same time, the model yields a more pronounced discrepancy in response times, which may be attributed to model limitations. In particular, the specific cell culture type (adherent versus suspension) was not incorporated into the framework. Furthermore, an increase in cell size is traditionally accompanied by enhanced transcription intensity. This is assumed to follow a dynamic equilibrium model, wherein global RNA polymerase II (RNAPII) transcription at a given cell size is dictated by the kinetics of recruiting free nucleoplasmic RNAPII to the genome according to the law of mass action [56]. However, the authors note that this transcriptional acceleration mechanism only provides a sublinear scaling of transcription relative to cell enlargement, which is further compensated for by a decrease in the mRNA degradation rate—a feature that was not accounted for in our current model.

In mammals, *BiP* is regulated by all three major arms of the UPR; thus, it receives complex regulatory transcriptional input. The *BiP* promoter contains three copies of the bipartite ER stress response element (ERSE), with a consensus sequence of CCAAT(N9)CCACG, located upstream of the TATA box [57]. *BiP* is regulated in an ERSE-dependent manner upon ER stress by i) the nuclear form of activating transcription factor 6 (pATF6(N)), along with YY1 and its interacting partners (the arginine methyltransferase PRMT1 and histone transacetylase p300), which bind to the CCACG box and enhance the binding of the multifunctional transcription factor TFII-I, or ii) the spliced form of *XBP1* [58]. Additionally, transcriptional activation of *BiP* can be mediated via ERSE-independent mechanisms through a complex involving ATF4 and its co-factors, ATF1 and CREB1 [59].

We have shown here that specific inhibition of the IRE1α ribonuclease domain leads to a marked reduction in BiP mRNA levels in Jurkat cells, whereas BiP mRNA expression even slightly increased in EA.hy926 cells. This result suggests a greater reliance on the IRE1α-XBP1 pathway for the regulation of BiP mRNA in Jurkat cells compared to EA.hy926 cells, which aligns with our bioinformatic analysis of the transcriptomic data for these two cell lines. In EA.hy926 cells, BiP expression is primarily determined by IRE1α-independent pathways, as corroborated by the continuous increase in BiP mRNA levels observed in endothelial cells despite the reduction in IRE1α-mediated XBP1 mRNA processing. This also accords with previous findings indicating that the level of the active nuclear form of ATF6 (pATF6(N)) in HEK293 cells decreases only 20 h after stress exposure, in contrast to IRE1α, which remains active for only 8 h [28].

XBP1 and ATF6 have previously been suggested to compensate for each other, at least in the transcription of the UPR genes Armet and Grp94 [59]. Taking into account that *BiP* is involved in the attenuation of XBP1-IRE1α signaling and that IRE1α participates in *BiP* mRNA degradation [60,61], our data indicate not only cooperative but also competitive interactions between IRE1α-dependent and IRE1α-independent regulatory inputs in controlling *BiP* mRNA levels under acute ER stress conditions.

The XBP1 gene is transcriptionally regulated by ATF6 and XBP1s, allowing it to regulate its own transcription as long as IRE1α remains active [62]. Our analysis shows that the temporal profile of IRE1α-XBP1s signaling is similar in the two studied cell lines. We attribute this effect to positive feedback within XBP1s signaling, which may provide a degree of temporal invariance to this pathway. In both exponentially growing and presenescent fibroblasts, we observed closely aligned initial segments of XBP1s temporal profiles, but a statistically significant longer response time in presenescent cells, reflecting prolonged XBP1s signaling and possibly failure in the feedback-mediated attenuation of IRE1α activity.

We observed a higher activation threshold for IRE1α-XBP1 signaling in EA.hy926 cells, likely due to their elevated basal BiP concentration. Although the abundance of RIDD-targeted mRNAs and the corresponding IRE1α enzymatic activity can differ significantly between high- and low-secreting cells, only minor differences in *XBP1u* mRNA splicing efficiency were detected between the two cell types. This is consistent with the lack of competition between RIDD and *XBP1* splicing observed in in vitro models of simultaneous *XBP1u* processing and RIDD activity [63].

It should be noted that utilizing a uniform concentration of the ER stress inducer across divergent cell lines does not strictly ensure an identical internal stress intensity within the ER lumen, as distinct cell types inevitably possess disparate susceptibility thresholds to reducing agents. At lower stress intensities, more sensitive T-lymphocytes initiate early signaling programs while a robust endothelium remains unresponsive. Crucially, as the concentration increases to the working benchmark, the stress becomes sufficient to overcome the baseline buffering capacity of the more resilient endothelial cells, bringing both models into an aligned functional activation dynamic for the primary pathways. Remarkably, our independent mathematical simulation for a small-ER compartment closely reproduces the empirical dynamic patterns of BiP at both the mRNA and protein levels of Jurkat cells. This qualitative alignment demonstrates that despite the higher susceptibility of T-cells to the reducing agent, their adaptive machinery remains functionally viable, even close to the uXBP1 mRNA splicing saturation threshold, confirming that spatial cellular architecture continues to exert a primary constraint on the BiP chaperone kinetics.

In our study, dithiothreitol (DTT) was selected as the ER stress inducer. As a potent reducing agent, DTT blocks the formation of disulfide bonds within the ER lumen [64] and rapidly initiates ER stress in cell cultures within minutes [65]. In eukaryotic cells, the oxidized environment of the ER is essential for the formation of most disulfide bonds [44], which are critical for the conformational maturation of proteins navigating the secretory pathway [49]. While the cytosolic redox balance is heavily shifted toward reduction, with a reduced-to-oxidized glutathione (GSH:GSSG) ratio of approximately 100:1 that prevents disulfide bonding, the ER lumen maintains a GSH:GSSG ratio of roughly 3:1 [66]. Although DTT can target disulfide-bonded proteins in various cellular compartments, such as extracellular domains, it predominantly impacts proteins en masse during their folding inside the ER, thereby triggering UPR signaling cascades, as previously demonstrated [45,53]. Tunicamycin, another canonical ER stress inducer, inhibits glycosylation and can interfere with the assaying of beta-galactosidase activity. Furthermore, due to the non-templated and multistage nature of protein glycosylation, its effects are inherently more challenging to incorporate into quantitative models compared to the stoichiometrically simpler and uniform disruption of disulfide bond formation by DTT. Nevertheless, the temporal dynamics of ER stress induced by alternative agents may exhibit unique cell type-specific features, which warrant further investigation.

In our study, presenescent FRSN cells were characterized by an enlarged size—a hallmark of cellular senescence first noted in the seminal works of Hayflick and Moorhead [67,68]—as well as by an expanded ER network. Traditionally, it has been assumed that organelle size and total protein mass scale proportionally with cell size, maintaining relatively constant protein concentrations. However, recent studies have revealed a more complex landscape, identifying proteins that are disproportionately scaled, either above or below the expected levels [50]. For instance, while cell cycle regulators often subscale, ER and lysosomal proteins have been identified as super-scaling [50]; notably, substantial expansion of the ER compartment was also confirmed in our experiments. Furthermore, mRNA scaling alone does not fully account for such proteomic behavior; incorporating codon frequency and post-transcriptional regulatory levels into models has been shown to improve the classification of protein scalability [50].

In our experiments with presenescent FRSN cells, while the BiP mRNA response times did not statistically differ from those of exponentially growing cells, we observed a significantly lower expression amplitude (relative to housekeeping genes) and a prolonged lag phase. If ER scaling occurs in these presenescent cells, the observed reduction in stress response mRNA and protein amplitudes may stem from global biosynthetic constraints, particularly the diminished DNA-to-protein ratio characteristic of enlarged, senescent cells [69,70,71].

Furthermore, we propose that the increase in cell size leads to a higher frequency of protein synthesis errors, necessitating the upregulation of folding and degradation systems, which could be manifested as the super-scaling of ER compartment proteins. Given that cell growth can be simplified as an exponential process [52], a corresponding exponential increase in the load of misfolded proteins is expected. The disruption of proteostasis acts as a fundamental limit to cell growth; reduced growth efficiency beyond a certain size threshold is a universal mechanism linked to a declining nucleic-acid-to-protein ratio (excluding cases of increased copy number, e.g., polyploidy). Excessively large cells are characterized by diminished mitochondrial potential [72], cytoplasmic dilution [55], and reduced proliferation [73]. Notably, as cell size increases, the temporal ‘tethering’ of the proteome to its genetic source (DNA and/or RNA) weakens, leading to less efficient protein turnover. Consequently, the cell may lose its regulatory precision, as expressed proteins may fail to reach their functional destinations at the required time, further exacerbating the collapse of proteostasis.

This discrepancy suggests that while the ER as an organelle may undergo super-scaling in size upon senescence, the stress response machinery fails to scale proportionally. This creates a state of ‘functional dilution’ within the enlarged ER, where the increased volume is not matched by a sufficient concentration of chaperones, ultimately leading to the protracted stress signaling and impaired proteostatic recovery frequently observed in senescent cells. This is consistent with previous reports of impaired inducible transcription in oversized cells [55].

Using scanning ion-conductance microscopy (SICM), we observed a decrease in the stiffness of senescent fibroblasts. This mechanical softening may be attributed to cytoplasmic dilution and the potential reduction in macromolecular crowding at least within the near-surface layer (the actin cortex), occurring as cell volume increases disproportionately to protein synthesis. While we cannot extrapolate these findings to the entire cytoplasmic volume, the observed changes in the cortical compartment suggest a transition toward a more fluid-like state in senescent cells. While cytoplasmic dilution potentially facilitates passive diffusion, it may impair the delivery of macromolecules—such as mRNAs and proteins—to their functional sites due to a decrease in the relative density of the cytoskeletal network. Consequently, despite a constant transport velocity, the probability of cargo reaching its destination (e.g., translation sites or the ER membrane) per unit of time significantly diminishes, extending the lag phase of the stress response.

## 5. Conclusions

We demonstrate that UPR dynamics are significantly modulated by cellular geometry. While the IRE1α-XBP1s pathway displays a high degree of temporal invariance across different backgrounds, BiP’s response kinetics appear closely correlated with the ER-to-cytoplasmic volume ratio. Under severe ER stress, cells with lower ER volumes exhibit accelerated BiP response times, which are driven by the rapid sequestration of limited free chaperone pools. In senescent cells, the disproportionate increase in volume and the rarefaction of the actin cortex (confirmed by SICM) lead to scaling failure of the stress response machinery and functional dilution of chaperones. This results in an extended lag phase and diminished stress response amplitude, rendering enlarged senescent cells more vulnerable to proteotoxic stress despite their persistent, protracted signaling profiles.

## Data Availability

The original contributions presented in this study are included in the article/Supplementary Material. Further inquiries can be directed to the corresponding author(s).

## Supplementary Materials

The following supporting information can be downloaded at: https://www.mdpi.com/article/10.3390/cimb48101027/s1.

## References

[1] Kozutsumi Y., Segal M., Normington K., Gething M.J., Sambrook J. (1988). The Presence of Malfolded Proteins in the Endoplasmic Reticulum Signals the Induction of Glucose-Regulated Proteins. Nature.

[2] Gardner B.M., Pincus D., Gotthardt K., Gallagher C.M., Walter P. (2013). Endoplasmic Reticulum Stress Sensing in the Unfolded Protein Response. Cold Spring Harb. Perspect. Biol..

[3] Schröder M., Kaufman R.J. (2005). The Mammalian Unfolded Protein Response. Annu. Rev. Biochem..

[4] Ron D., Walter P. (2007). Signal Integration in the Endoplasmic Reticulum Unfolded Protein Response. Nat. Rev. Mol. Cell Biol..

[5] Travers K.J., Patil C.K., Wodicka L., Lockhart D.J., Weissman J.S., Walter P. (2000). Functional and Genomic Analyses Reveal an Essential Coordination between the Unfolded Protein Response and ER-Associated Degradation. Cell.

[6] Harding H.P., Zhang Y., Ron D. (1999). Protein Translation and Folding Are Coupled by an Endoplasmic-Reticulum-Resident Kinase. Nature.

[7] Hollien J., Lin J.H., Li H., Stevens N., Walter P., Weissman J.S. (2009). Regulated Ire1-Dependent Decay of Messenger RNAs in Mammalian Cells. J. Cell Biol..

[8] Mesitov M.V., Moskovtsev A.A., Kubatiev A.A. (2013). Molecular logic of the endoplasmic reticulum stress signal pathways: The system of unfolded protein response. Patol. Fiziol. Eksp. Ter..

[9] Lee K., Tirasophon W., Shen X., Michalak M., Prywes R., Okada T., Yoshida H., Mori K., Kaufman R.J. (2002). IRE1-Mediated Unconventional mRNA Splicing and S2P-Mediated ATF6 Cleavage Merge to Regulate XBP1 in Signaling the Unfolded Protein Response. Genes Dev..

[10] Nadanaka S., Okada T., Yoshida H., Mori K. (2007). Role of Disulfide Bridges Formed in the Luminal Domain of ATF6 in Sensing Endoplasmic Reticulum Stress. Mol. Cell Biol..

[11] Ye J., Rawson R.B., Komuro R., Chen X., Davé U.P., Prywes R., Brown M.S., Goldstein J.L. (2000). ER Stress Induces Cleavage of Membrane-Bound ATF6 by the Same Proteases That Process SREBPs. Mol. Cell.

[12] Ranawade A., Sharma R., Levine E. (2022). Lessons Learned from Two Decades of Modeling the Heat-Shock Response. Biomolecules.

[13] Schindler A.J., Schekman R. (2009). In Vitro Reconstitution of ER-Stress Induced ATF6 Transport in COPII Vesicles. Proc. Natl. Acad. Sci. USA.

[14] Ma K., Vattem K.M., Wek R.C. (2002). Dimerization and Release of Molecular Chaperone Inhibition Facilitate Activation of Eukaryotic Initiation Factor-2 Kinase in Response to Endoplasmic Reticulum Stress. J. Biol. Chem..

[15] Jousse C., Deval C., Maurin A.-C., Parry L., Chérasse Y., Chaveroux C., Lefloch R., Lenormand P., Bruhat A., Fafournoux P. (2007). TRB3 Inhibits the Transcriptional Activation of Stress-Regulated Genes by a Negative Feedback on the ATF4 Pathway. J. Biol. Chem..

[16] Vattem K.M., Wek R.C. (2004). Reinitiation Involving Upstream ORFs Regulates ATF4 mRNA Translation in Mammalian Cells. Proc. Natl. Acad. Sci. USA.

[17] Trusina A., Papa F.R., Tang C. (2008). Rationalizing Translation Attenuation in the Network Architecture of the Unfolded Protein Response. Proc. Natl. Acad. Sci. USA.

[18] Pincus D., Chevalier M.W., Aragón T., van Anken E., Vidal S.E., El-Samad H., Walter P. (2010). BiP Binding to the ER-Stress Sensor Ire1 Tunes the Homeostatic Behavior of the Unfolded Protein Response. PLoS Biol..

[19] Erguler K., Pieri M., Deltas C. (2013). A Mathematical Model of the Unfolded Protein Stress Response Reveals the Decision Mechanism for Recovery, Adaptation and Apoptosis. BMC Syst. Biol..

[20] Onn A., Ron D. (2010). Modeling the Endoplasmic Reticulum Unfolded Protein Response. Nat. Struct. Mol. Biol..

[21] Hutt D.M., Balch W.E. (2013). Expanding Proteostasis by Membrane Trafficking Networks. Cold Spring Harb. Perspect. Biol..

[22] Ma Y., Hendershot L.M. (2004). ER Chaperone Functions during Normal and Stress Conditions. J. Chem. Neuroanat..

[23] Hebert D.N., Molinari M. (2007). In and out of the ER: Protein Folding, Quality Control, Degradation, and Related Human Diseases. Physiol. Rev..

[24] Zhang J.-X., Braakman I., Matlack K.E.S., Helenius A. (1997). Quality Control in the Secretory Pathway: The Role of Calreticulin, Calnexin and BiP in the Retention of Glycoproteins with C-Terminal Truncations. Mol. Biol. Cell.

[25] Korennykh A.V., Egea P.F., Korostelev A.A., Finer-Moore J., Zhang C., Shokat K.M., Stroud R.M., Walter P. (2009). The Unfolded Protein Response Signals through High-Order Assembly of Ire1. Nature.

[26] Korennykh A.V., Korostelev A.A., Egea P.F., Finer-Moore J., Stroud R.M., Zhang C., Shokat K.M., Walter P. (2011). Structural and Functional Basis for RNA Cleavage by Ire1. BMC Biol..

[27] Lin J.H., Li H., Yasumura D., Cohen H.R., Zhang C., Panning B., Shokat K.M., Lavail M.M., Walter P. (2007). IRE1 Signaling Affects Cell Fate during the Unfolded Protein Response. Science.

[28] Bertolotti A., Zhang Y., Hendershot L.M., Harding H.P., Ron D. (2000). Dynamic Interaction of BiP and ER Stress Transducers in the Unfolded-Protein Response. Nat. Cell Biol..

[29] Eletto D., Eletto D., Dersh D., Gidalevitz T., Argon Y. (2014). Protein Disulfide Isomerase A6 Controls the Decay of IRE1α Signaling via Disulfide-Dependent Association. Mol. Cell.

[30] Mesitov M.V., Ignashkova T.I., Meshcherskiĭ M.E., Akopov A.S., Sokolovskaia A.A., Moskovtsev A.A., Kubatiev A.A. (2012). Induction of the endoplasmic reticulum stress in conditions of acid-base imbalance in human cells of T-lymphoblastic leukemia. Patol. Fiziol. Eksp. Ter..

[31] Hetz C. (2012). The Unfolded Protein Response: Controlling Cell Fate Decisions under ER Stress and Beyond. Nat. Rev. Mol. Cell Biol..

[32] Poljanskaya G., Bobkov D., Koltsova A., Musorina A., Mikhailova N. (2022). Creation, working principles, development of applied and fundamental scientific activities of the Collection of Cell Cultures of Vertebrates. Biol. Commun..

[33] Ignashkova T.I., Mesitov M.V., Rybakov A.S., Moskovtsev A.A., Sokolovskaia A.A., Kubatiev A.A. (2012). Deposition of von Willebrand factor in human endothelial cells HUVEC in the endoplasmic reticulum stress induced by an excess of homocysteine in vitro. Patol. Fiziol. Eksp. Ter..

[34] SRP323222—SRA—NCBI. https://www.ncbi.nlm.nih.gov/sra?term=SRP323222.

[35] Ghandi M., Huang F.W., Jané-Valbuena J., Kryukov G.V., Lo C.C., McDonald E.R., Barretina J., Gelfand E.T., Bielski C.M., Li H. (2019). Next-Generation Characterization of the Cancer Cell Line Encyclopedia. Nature.

[36] Mootha V.K., Lindgren C.M., Eriksson K.-F., Subramanian A., Sihag S., Lehar J., Puigserver P., Carlsson E., Ridderstråle M., Laurila E. (2003). PGC-1α-Responsive Genes Involved in Oxidative Phosphorylation Are Coordinately Downregulated in Human Diabetes. Nat. Genet..

[37] Subramanian A., Tamayo P., Mootha V.K., Mukherjee S., Ebert B.L., Gillette M.A., Paulovich A., Pomeroy S.L., Golub T.R., Lander E.S. (2005). Gene Set Enrichment Analysis: A Knowledge-Based Approach for Interpreting Genome-Wide Expression Profiles. Proc. Natl. Acad. Sci. USA.

[38] Hirota M., Kitagaki M., Itagaki H., Aiba S. (2006). Quantitative Measurement of Spliced XBP1 mRNA as an Indicator of Endoplasmic Reticulum Stress. J. Toxicol. Sci..

[39] Cohen J. (2013). Statistical Power Analysis for the Behavioral Sciences.

[40] Chechik G., Koller D. (2009). Timing of Gene Expression Responses to Environmental Changes. J. Comput Biol..

[41] Chechik G., Oh E., Rando O., Weissman J., Regev A., Koller D. (2008). Activity Motifs Reveal Principles of Timing in Transcriptional Control of the Yeast Metabolic Network. Nat. Biotechnol..

[42] Yosef N., Regev A. (2011). Impulse Control: Temporal Dynamics in Gene Transcription. Cell.

[43] Holloway E.M., Wu J.H., Czerwinkski M., Sweet C.W., Wu A., Tsai Y.-H., Huang S., Stoddard A.E., Capeling M.M., Glass I. (2020). Differentiation of Human Intestinal Organoids with Endogenous Vascular Endothelial Cells. Dev. Cell.

[44] Haze K., Yoshida H., Yanagi H., Yura T., Mori K. (1999). Mammalian Transcription Factor ATF6 Is Synthesized as a Transmembrane Protein and Activated by Proteolysis in Response to Endoplasmic Reticulum Stress. Mol. Biol. Cell.

[45] Mesitov M.V., Soldatov R.A., Zaichenko D.M., Malakho S.G., Klementyeva T.S., Sokolovskaya A.A., Kubatiev A.A., Mironov A.A., Moskovtsev A.A. (2017). Differential Processing of Small RNAs during Endoplasmic Reticulum Stress. Sci. Rep..

[46] Papandreou I., Denko N.C., Olson M., Van Melckebeke H., Lust S., Tam A., Solow-Cordero D.E., Bouley D.M., Offner F., Niwa M. (2011). Identification of an Ire1alpha Endonuclease Specific Inhibitor with Cytotoxic Activity against Human Multiple Myeloma. Blood.

[47] Marciniak S.J., Yun C.Y., Oyadomari S., Novoa I., Zhang Y., Jungreis R., Nagata K., Harding H.P., Ron D. (2004). CHOP Induces Death by Promoting Protein Synthesis and Oxidation in the Stressed Endoplasmic Reticulum. Genes Dev..

[48] Bernales S., Papa F.R., Walter P. (2006). Intracellular Signaling by the Unfolded Protein Response. Annu. Rev. Cell Dev. Biol..

[49] Berenson D.F., Zatulovskiy E., Xie S., Skotheim J.M. (2019). Constitutive Expression of a Fluorescent Protein Reports the Size of Live Human Cells. Mol. Biol. Cell.

[50] Lanz M.C., Zatulovskiy E., Swaffer M.P., Zhang L., Ilerten I., Zhang S., You D.S., Marinov G., McAlpine P., Elias J.E. (2022). Increasing Cell Size Remodels the Proteome and Promotes Senescence. Mol. Cell.

[51] Chan Y.-H.M., Marshall W.F. (2010). Scaling Properties of Cell and Organelle Size. Organogenesis.

[52] Lin J., Amir A. (2018). Homeostasis of Protein and mRNA Concentrations in Growing Cells. Nat. Commun..

[53] Zaichenko D.M., Mikryukova A.A., Astafeva I.R., Malakho S.G., Kubatiev A.A., Moskovtsev A.A. (2023). MicroRNA Biogenesis in Cell Senescence Induced by Chronic Endoplasmic Reticulum Stress. Mol. Biol..

[54] Gorelik J., Shevchuk A.I., Frolenkov G.I., Diakonov I.A., Lab M.J., Kros C.J., Richardson G.P., Vodyanoy I., Edwards C.R.W., Klenerman D. (2003). Dynamic Assembly of Surface Structures in Living Cells. Proc. Natl. Acad. Sci. USA.

[55] Neurohr G.E., Terry R.L., Lengefeld J., Bonney M., Brittingham G.P., Moretto F., Miettinen T.P., Vaites L.P., Soares L.M., Paulo J.A. (2019). Excessive Cell Growth Causes Cytoplasm Dilution And Contributes to Senescence. Cell.

[56] Swaffer M.P., Marinov G.K., Zheng H., Fuentes Valenzuela L., Tsui C.Y., Jones A.W., Greenwood J., Kundaje A., Greenleaf W.J., Reyes-Lamothe R. (2023). RNA Polymerase II Dynamics and mRNA Stability Feedback Scale mRNA Amounts with Cell Size. Cell.

[57] Yoshida H., Haze K., Yanagi H., Yura T., Mori K. (1998). Identification of the Cis-Acting Endoplasmic Reticulum Stress Response Element Responsible for Transcriptional Induction of Mammalian Glucose-Regulated Proteins. Involvement of Basic Leucine Zipper Transcription Factors. J. Biol. Chem..

[58] Lee A.-H., Iwakoshi N.N., Glimcher L.H. (2003). XBP-1 Regulates a Subset of Endoplasmic Reticulum Resident Chaperone Genes in the Unfolded Protein Response. Mol. Cell Biol..

[59] Luo S., Baumeister P., Yang S., Abcouwer S.F., Lee A.S. (2003). Induction of Grp78/BiP by Translational Block: Activation of the Grp78 Promoter by ATF4 through and Upstream ATF/CRE Site Independent of the Endoplasmic Reticulum Stress Elements. J. Biol. Chem..

[60] Han D., Lerner A.G., Vande Walle L., Upton J.-P., Xu W., Hagen A., Backes B.J., Oakes S.A., Papa F.R. (2009). IRE1alpha Kinase Activation Modes Control Alternate Endoribonuclease Outputs to Determine Divergent Cell Fates. Cell.

[61] Yoshida H., Matsui T., Yamamoto A., Okada T., Mori K. (2001). XBP1 mRNA Is Induced by ATF6 and Spliced by IRE1 in Response to ER Stress to Produce a Highly Active Transcription Factor. Cell.

[62] Oikawa D., Tokuda M., Hosoda A., Iwawaki T. (2010). Identification of a Consensus Element Recognized and Cleaved by IRE1 Alpha. Nucleic Acids Res..

[63] Li H., Korennykh A.V., Behrman S.L., Walter P. (2010). Mammalian Endoplasmic Reticulum Stress Sensor IRE1 Signals by Dynamic Clustering. Proc. Natl. Acad. Sci. USA.

[64] Braakman I., Helenius J., Helenius A. (1992). Manipulating Disulfide Bond Formation and Protein Folding in the Endoplasmic Reticulum. EMBO J..

[65] Oslowski C.M., Urano F. (2011). Measuring ER Stress and the Unfolded Protein Response Using Mammalian Tissue Culture System. Methods Enzymol..

[66] Hwang C., Sinskey A.J., Lodish H.F. (1992). Oxidized Redox State of Glutathione in the Endoplasmic Reticulum. Science.

[67] Hayflick L., Moorhead P.S. (1961). The Serial Cultivation of Human Diploid Cell Strains. Exp. Cell Res..

[68] Hayflick L. (1965). The Limited in Vitro Lifetime of Human Diploid Cell Strains. Exp. Cell Res..

[69] Zhurinsky J., Leonhard K., Watt S., Marguerat S., Bähler J., Nurse P. (2010). A Coordinated Global Control over Cellular Transcription. Curr. Biol..

[70] Elliott S.G. (1983). Regulation of the Maximal Rate of RNA Synthesis in the Fission Yeast Schizosaccharomyces Pombe. Mol. Gen. Genet..

[71] Swaffer M.P., Kim J., Chandler-Brown D., Langhinrichs M., Marinov G., Greenleaf W., Kundaje A., Schmoller K.M., Skotheim J.M. (2021). Transcriptional and Chromatin-Based Partitioning Mechanisms Uncouple Protein Scaling from Cell Size. Mol. Cell.

[72] Miettinen T.P., Björklund M. (2016). Cellular Allometry of Mitochondrial Functionality Establishes the Optimal Cell Size. Dev. Cell.

[73] Demidenko Z.N., Blagosklonny M.V. (2008). Growth Stimulation Leads to Cellular Senescence When the Cell Cycle Is Blocked. Cell Cycle.

