# Supplementary material for: Dynamics of Endoplasmic Reticulum Stress Responses in Cells with Different Endoplasmic Reticulum Volumes and Senescence States
Source: Curr Issues Mol Biol. 2026 Oct 4;48(10):1027. doi: 10.3390/cimb48101027 (PMC13703400; doi:10.3390/cimb48101027)
Supplement: Supplementary file 1 [file cimb-48-01027-s001.zip › cimb-4535787-supplementary.pdf]

current issues in molecular biology

MDPI

Supplementary

Supplementary Table S1. Gene sets enriched in EA.hy926 cells at an FDR threshold of < 25%.

NAME	SIZE	NES	NOM p-val	FDR q-val
SYNAPTIC CLEFT	16	1.51	0.00	0.17
BLOOD MICROPARTICLE	64	1.52	0.00	0.17
CORTICAL CYTOSKELETON	89	1.43	0.00	0.18
GOLGI LUMEN	58	1.43	0.01	0.18
BASEMENT MEMBRANE	80	1.43	0.00	0.18
EXTERNAL ENCAPSULATING STRUCTURE	309	1.43	0.00	0.18
I BAND	106	1.43	0.00	0.19
TERTIARY GRANULE LUMEN	38	1.43	0.03	0.20
VESICLE LUMEN	254	1.43	0.00	0.20
CYTOSOLIC LARGE RIBOSOMAL SUBUNIT	54	1.41	0.01	0.21
DENDRITIC SHAFT	34	1.40	0.03	0.21
PLATELET ALPHA GRANULE MEMBRANE	15	1.45	0.01	0.21
CATENIN COMPLEX	18	1.40	0.04	0.21
CORTICAL ACTIN CYTOSKELETON	65	1.40	0.01	0.21
ENDOLYSOSOME	27	1.41	0.03	0.21
H4 H2A HISTONE ACETYLTRANSFERASE COMPLEX	24	1.42	0.03	0.21
CELL SUBSTRATE JUNCTION	384	1.41	0.00	0.21
PIGMENT GRANULE	105	1.41	0.00	0.21
FICOLIN 1 RICH GRANULE	152	1.43	0.00	0.21
RUFFLE MEMBRANE	90	1.40	0.01	0.21
SCHAFFER COLLATERAL CA1 SYNAPSE	69	1.41	0.02	0.21
SECONDARY LYSOSOME	23	1.48	0.01	0.21
LAMELLIPODIUM MEMBRANE	21	1.45	0.03	0.21
MYELIN SHEATH	33	1.35	0.06	0.22
CONTRACTILE MUSCLE FIBER	175	1.43	0.00	0.22
PROTEIN LIPID COMPLEX	19	1.45	0.02	0.22
ENDOSOME LUMEN	26	1.41	0.04	0.22
CELL LEADING EDGE	372	1.36	0.00	0.22
AXONEMAL MICROTUBULE	22	1.41	0.03	0.22
LARGE RIBOSOMAL SUBUNIT	108	1.35	0.02	0.22
PROTEIN COMPLEX INVOLVED IN CELL MATRIX ADHESION	16	1.41	0.03	0.22
FILAMENTOUS ACTIN	25	1.44	0.01	0.22
ROUGH ENDOPLASMIC RETICULUM	58	1.34	0.06	0.22
SPECIFIC GRANULE LUMEN	41	1.35	0.04	0.22
APICOLATERAL PLASMA MEMBRANE	16	1.36	0.05	0.22
PODOSOME	36	1.44	0.02	0.22
CELL PROJECTION MEMBRANE	260	1.34	0.00	0.22
CAVEOLA	65	1.35	0.03	0.22
T TUBULE	32	1.34	0.06	0.22
FICOLIN 1 RICH GRANULE MEMBRANE	40	1.35	0.06	0.22
INTEGRIN COMPLEX	27	1.34	0.06	0.22
NUCLEOSOME	78	1.36	0.03	0.22
ENDOPLASMIC RETICULUM GOLGI INTERMEDIATE COMPARTMENT	119	1.35	0.01	0.22
PLATELET DENSE GRANULE	15	1.33	0.08	0.22
PLATELET ALPHA GRANULE LUMEN	47	1.49	0.00	0.22
ACTIN FILAMENT BUNDLE	82	1.36	0.03	0.22
CYTOPLASMIC SIDE OF MEMBRANE	181	1.34	0.00	0.22
CELL CELL CONTACT ZONE	56	1.33	0.06	0.22
LIPID DROPLET	81	1.36	0.02	0.22
CLATHRIN COAT OF COATED PIT	16	1.35	0.06	0.22
LAMELLIPODIUM	182	1.34	0.00	0.23
EXTERNAL SIDE OF PLASMA MEMBRANE	207	1.33	0.00	0.23
SMOOTH ENDOPLASMIC RETICULUM	20	1.37	0.06	0.23
AZUROPHIL GRANULE	128	1.35	0.01	0.23
AZUROPHIL GRANULE LUMEN	74	1.33	0.04	0.23
EXTRINSIC COMPONENT OF PLASMA MEMBRANE	71	1.36	0.03	0.23
CALCIUM CHANNEL COMPLEX	43	1.34	0.05	0.23
AZUROPHIL GRANULE MEMBRANE	48	1.36	0.04	0.23
ACTIN FILAMENT	99	1.37	0.02	0.23

PROTEIN COMPLEX INVOLVED IN CELL ADHESION	48	1.43	0.01	0.23
MYOFILAMENT	15	1.33	0.07	0.23
NUCLEAR OUTER MEMBRANE	28	1.33	0.08	0.23
SARCOPLASM	62	1.36	0.03	0.23
VACUOLAR LUMEN	134	1.36	0.01	0.23
PLATELET ALPHA GRANULE	69	1.48	0.00	0.23
GAMMA TUBULIN COMPLEX	18	1.33	0.07	0.23
INTERSTITIAL MATRIX	18	1.33	0.06	0.23
LEADING EDGE MEMBRANE	145	1.37	0.00	0.23
TIGHT JUNCTION	104	1.36	0.01	0.23
VOLTAGE GATED CALCIUM CHANNEL COMPLEX	24	1.33	0.08	0.23
RUFFLE	170	1.38	0.00	0.23
KERATIN FILAMENT	19	1.45	0.00	0.23
NEUROMUSCULAR JUNCTION	68	1.37	0.02	0.23
ENDOPLASMIC RETICULUM LUMEN	233	1.39	0.00	0.23
PROTON TRANSPORTING TWO SECTOR ATPASE COMPLEX PROTON TRANSPORTING DOMAIN	15	1.33	0.09	0.23
ENDOPLASMIC RETICULUM GOLGI INTERMEDIATE COMPARTMENT MEMBRANE	70	1.38	0.03	0.23
CASPASE COMPLEX	15	1.38	0.05	0.23
CELL CELL JUNCTION	398	1.36	0.00	0.23
ACTIN CYTOSKELETON	395	1.37	0.00	0.23
H4 HISTONE ACETYLTRANSFERASE COMPLEX	37	1.38	0.03	0.23
APICAL JUNCTION COMPLEX	118	1.38	0.00	0.23
BRUSH BORDER	68	1.37	0.02	0.24
SARCOLEMMA	96	1.39	0.00	0.24
NEURON PROJECTION TERMINUS	73	1.38	0.02	0.24
ADHERENS JUNCTION	161	1.36	0.00	0.24
ACROSOMAL MEMBRANE	23	1.38	0.06	0.24
LUMENAL SIDE OF MEMBRANE	34	1.32	0.06	0.24
APICAL DENDRITE	15	1.39	0.04	0.24
LYSOSOMAL LUMEN	72	1.38	0.02	0.24
SIDE OF MEMBRANE	459	1.36	0.00	0.24
CELL CORTEX	272	1.37	0.00	0.24
GLYCOPROTEIN COMPLEX	16	1.49	0.01	0.24
CORNIFIED ENVELOPE	26	1.48	0.01	0.24

Supplementary Table S2. Gene sets enriched in Jurkat cells.

NAME	SIZE	NES	NOM p-val	FDR q-val
NADH DEHYDROGENASE COMPLEX	46	-1.73	0.09	0.36
PROTON TRANSPORTING TWO SECTOR ATPASE COMPLEX	46	-1.78	0.05	0.38
PROTON TRANSPORTING ATP SYNTHASE COMPLEX	20	-1.85	0.03	0.41
REPLISOME	24	-1.28	0.23	0.48
OXIDOREDUCTASE COMPLEX	105	-1.29	0.25	0.50
MONOATOMIC ION CHANNEL COMPLEX	192	-1.23	0.21	0.53
CATION CHANNEL COMPLEX	124	-1.30	0.18	0.54
RESPIRATORY CHAIN COMPLEX IV	18	-1.34	0.17	0.58
TRANSPORTER COMPLEX	344	-1.45	0.01	0.58
RESPIRATORY CHAIN COMPLEX	104	-1.41	0.09	0.59
PERICENTRIOLAR MATERIAL	23	-1.30	0.15	0.59
CYTOCHROME COMPLEX	33	-1.36	0.15	0.60
INNER KINETOCHORE	17	-1.46	0.08	0.67
DNA POLYMERASE COMPLEX	19	-1.02	0.42	0.70
NUCLEAR EXOSOME RNASE COMPLEX	16	-0.97	0.48	0.71
OUTER KINETOCHORE	20	-1.50	0.09	0.71
NUCLEAR REPLICATION FORK	44	-1.06	0.38	0.74
GABA ERGIC SYNAPSE	62	-1.02	0.39	0.74
CHROMATIN SILENCING COMPLEX	18	-0.98	0.43	0.74
TRANSCRIPTION FACTOR TFIID COMPLEX	31	-1.08	0.29	0.76
AXONEMAL DOUBLET MICROTUBULE	18	-1.03	0.38	0.77
REPLICATION FORK	77	-0.92	0.42	0.78
ESC E Z COMPLEX	16	-0.89	0.58	0.79
U1 SNRNP	20	-0.86	0.59	0.82
SMN SM PROTEIN COMPLEX	19	-0.84	0.65	0.83

https://doi.org/10.3390/xxxxx

DNA REPAIR COMPLEX	23	-0.81	0.65	0.85
PHOTORECEPTOR CONNECTING CILIUM	41	-0.70	0.74	0.91
CAJAL BODY	61	-0.72	0.87	0.92
NUCLEAR CHROMOSOME	214	-0.67	0.98	0.93
MICROBODY LUMEN	40	-0.64	0.91	0.94
SNO S RNA CONTAINING RIBONUCLEOPROTEIN COMPLEX	25	-0.72	0.74	0.95
CONDENSED NUCLEAR CHROMOSOME	61	-0.74	0.67	0.95
NUCLEAR CYCLIN DEPENDENT PROTEIN KINASE HOLOENZYME COMPLEX	26	-0.60	0.94	0.95
